# Exploring the Concept of Nurses’ Healthcare Policy Competence: A Systematic Review and Qualitative Meta‐Synthesis

**DOI:** 10.1155/jonm/9182331

**Published:** 2026-03-20

**Authors:** Nam Kyung Han, Jong Gun Kim

**Affiliations:** ^1^ Department of Nursing, Gyeongbuk College of Health, Daehakro 168, Gimcheon, 39525, Gyeongbuk, South Korea; ^2^ Department of Nursing Science, Hoseo University, 79 Hoseo-ro 20beon-gil Baebang-eup, Asan-si, 31499, Chungnam, South Korea, hoseo.ac.kr

**Keywords:** competence, healthcare, meta-synthesis, nurses, policy, qualitative

## Abstract

**Background:**

Nurses’ competence in healthcare policy is crucial for policy intervention to strengthen public health equity and resolve health problems through healthcare policy reforms. Previous studies conducted systematic reviews, scoping reviews, or meta‐syntheses related to the political competence of nurses, factors affecting nurses’ policy interventions, and barriers and facilitators of policy intervention, but the concept of healthcare policy competence was not examined.

**Aim:**

This study explored nurses’ competence in healthcare policy through a systematic review and meta‐synthesis of previous qualitative studies.

**Methods:**

A systematic review of English‐language journal articles in databases including Medline, Embase, PubMed, CINAHL, Web of Science, and Scopus published up to December 31, 2023, generated 17,810 identified articles, of which 27 individual studies were used for analysis. Researchers with rich experiences in healthcare policy interventions and qualitative research analysis extracted data using ATLAS.ti and meta‐synthesized it.

**Results:**

Nurses’ competence in healthcare policy was synthesized into five factors: intrinsic motivation, healthcare expertise, policy development, political skills, and policy intervention. Fifteen themes and 34 subthemes were derived as subattributes.

**Conclusions:**

This study provides a conceptual framework for nurses’ competence in healthcare policy and for developing political or policy education or programs to strengthen this competence.

## 1. Introduction

The healthcare sector is a more specific and crucial policy sector than other fields that directly affects people’s lives. Hence, healthcare policy reforms must keep pace with evolving healthcare environments [1]. In this regard, most countries worldwide face changes in their healthcare environments, including population aging, the continued rise in noncommunicable diseases, and the emergence of new infectious diseases such as coronavirus disease 2019 [2]. Therefore, each country must strengthen health equity and preventive public health management and resolve health problems through healthcare policy reforms in response to changing healthcare environments that may threaten future health or cause new public health crises [2, 3].

The policy is defined as an authoritative allocation of values for society [4] or a forward‐looking basic process of action formally determined by government agencies through a complex and dynamic process [5, 6]. The process for developing public policy includes agenda setting, policy formulation, adoption, implementation, and evaluation [7, 8]. It also involves a wide range of healthcare professionals and stakeholders. The process is dynamic, political, and unpredictable, often resulting in public interest being ignored and policy reforms drifting or failing [9]. Healthcare‐related policymaking and legislative processes are shaped by diverse and sharp interests and structures among parliamentarians who participate in enacting and amending health‐related legislation in the National Assembly and the healthcare professionals who influence their decision‐making [10]. Therefore, the competence of nurses in healthcare policy, including political skills, is essential for policy intervention and legislation. Comprising approximately 59.0% of the healthcare profession, nursing professionals are integral to the healthcare systems of most countries and can be crucial for achieving healthcare policy reforms [3]. Nevertheless, the healthcare policy competence of nurses is considered insufficient because most nurses lack opportunities to participate in the healthcare policy formation process and do not adequately address policy agendas [11–14].

Policy competence is the ability to gather necessary resources [15], considering the organizational diversity and interests of governments [16]. Healthcare policy competence has the intrinsic motivational attributes, including awareness of health issues or problems, social responsibility, and sustained commitment to healthcare policymaking [17–19]. It is also the ability to understand the political dynamics and healthcare policy process, beliefs, strategies, and interactions of relevant politicians or policymakers and stakeholders and to use effective and diverse political skills [13, 20]. In addition, healthcare policy competence includes the ability to engage in policy interventions by developing policy alternatives and shaping or leading public opinions [12, 20].

Previous studies that conducted systematic reviews, scoping reviews, or meta‐syntheses related to nurses’ healthcare policy competence include Bing‐Jonsson et al. [21], Azimirad et al. [22], Souza et al. [23], Benton et al. [24], Hajizadeh et al. [18], and Han and Kim [11]. Among these, Bing‐Jonsson et al. [21] and Azimirad et al. [22] focused on the competence of community health nursing practice, and Souza et al. [23] focused on the competence of public health nursing practice. Hence, they had little relevance to the synthesis of competence in healthcare policy. Previous studies related to this study included Benton et al. [24], Hajizadeh et al. [18], and Han and Kim [11]. Among them, Benton et al. [24] conducted an integrative review of the current status of studies on nurses’ political competence and policy pursuit. Hajizadeh et al. [18] conducted a systematic review by dividing the factors and frameworks on the influence of nurses’ participation in the healthcare policy decision‐making process into three factors: nursing‐related, management and organizational, and work environments. Han and Kim [11] conducted a systematic review and meta‐synthesis of the barriers and facilitators of nurses’ policy intervention. Despite this extensive research, no study has conducted a systematic review and meta‐synthesis on nurses’ competence in healthcare policy. Therefore, this study aims to explore the concept of nurses’ competence in healthcare policy through a systematic review and meta‐synthesis of qualitative previous studies.

## 2. Methods

### 2.1. Research Design

This study was a qualitative meta‐synthesis to derive the concept of nurses’ competence in healthcare policy based on Noblit and Hare’s [25] meta‐ethnography, which provides comprehensive insight into research or social phenomena. Qualitative meta‐synthesis overcomes the limitations of generalizing individual qualitative studies by interpreting the qualitative research results. The design derives accumulated knowledge and new interpretations of the research phenomenon [26–28] and enables the development of new theory through higher levels of abstraction and generalization [29].

The primary qualitative meta‐synthesis methods include meta‐ethnography [25], critical interpretive synthesis [30], grounded theory synthesis [31], thematic synthesis [32], and meta‐study [33]. Among these, meta‐ethnography helps generate new interpretations through comparison and analysis in various fields, including health‐related fields. Therefore, it is used widely as a qualitative meta‐synthesis method. Accordingly, the meta‐ethnography meta‐synthesis method was used because it was the most appropriate for this study. This study was conducted in accordance with the meta‐analysis protocol registered in PROSPERO, the International Prospective Register of Systematic Reviews (Registration ID: CRD42022346992). This study utilized existing datasets and was exempt from ethics approval. This study explored the concepts of nurses’ competence in healthcare policy.

### 2.2. Search Strategy and Study Selection

The study selection for analysis was based on the Preferred Reporting Items for Systematic Reviews and Meta‐Analyses (PRISMA) 2020 flowchart [34]. The PRISMA 2020 guideline is an international standard for meta‐synthesis consisting of a 27‐item checklist that provides research credibility and transparency. The participants in the individual studies included in this study were nurses, and the phenomena of interest were previous studies on nurses’ healthcare policy competence. The search was conducted from September 5, 2023, to March 30, 2024, and included qualitative studies and doctoral dissertations published in English in journals in the database until December 31, 2023. The scope of qualitative studies included ethnography, grounded theory, phenomenology, and qualitative descriptive studies that described the participants’ statements. The inclusion criteria included participants from the following groups with extensive experiences in healthcare policy intervention or political activism: nurses, registered nurses, licensed nurses, the nursing profession, nursing professionals, and midwives. The exclusion criteria included non‐nurses, nursing personnel or staff not including nursing professionals, other healthcare professionals, nursing students, quantitative studies, perspectives, and commentaries (Supporting Table 1).

Before the systematic review, researchers independently conducted a preliminary search to identify the main keywords and other relevant information and establish a search strategy. The databases used in this study were Medline, Embase, PubMed, CINAHL, Web of Science, and Scopus based on the COre search electronic databases of COSI (COre, Standard, Ideal) by the National Library of Medicine. In addition, a manual search was conducted on Google Scholar. The search strategy was Nurse^∗^ OR Licensed nurse^∗^ OR Registered nurse^∗^ OR Nursing profession OR Nursing professional OR midwives OR midwifery OR midwife AND Politics OR Political OR Policy OR Policies OR Advocac^∗^ AND Legislat^∗^ OR Public health OR Advocac^∗^ AND Competenc^∗^ OR Experience^∗^ OR Capacit^∗^ OR Capabilit^∗^ OR Leadership OR abilit^∗^ OR Socialization OR Socialisation AND Qualitative^∗^ OR Interview^∗^ OR Empirical OR Fieldwork OR Field work OR Focus group^∗^ OR Unstructured OR In‐depth OR Semi‐structured OR Indepth OR Face‐to‐face OR Phenomenolog^∗^ OR Grounded theory OR Ethnograph^∗^ (Supporting Table 2). Two researchers conducted the literature search independently, and the titles, abstracts, and full texts were reviewed after duplicate studies were removed using EndNote 20. In addition, a consensus was reached on any inconsistencies after sufficient discussion between the two researchers.

### 2.3. Characteristics of the Selected Studies and Quality Assessment

**FIGURE 1:**
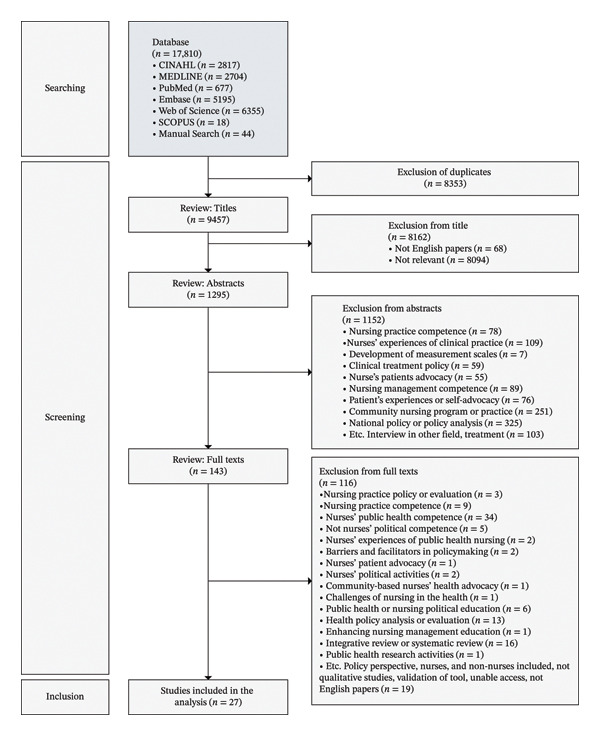
Study selection process using PRISMA 2020 [24].

Database and manual searches identified 17,810 studies. After the initial search, 8353 duplicate articles were excluded, and 9457 titles were reviewed. After excluding 68 non‐English articles and 8094 articles irrelevant to this study, the abstracts of 1295 articles were reviewed. A total of 1152 articles that did not meet the selection criteria were excluded, and the full texts of the remaining 143 articles were reviewed. In the process, 116 articles on nursing practice competence, nursing policy evaluation, public health practice competence, political education, and policy analysis were excluded, leaving 27 individual studies for meta‐synthesis analysis (Figure 1) (Supporting Table 3).

Twenty‐seven individual studies were included in the final analysis from the following ten countries: USA (*n* = 12), Iran (*n* = 4), Canada (*n* = 4), New Zealand (*n* = 1), South Korea (*n* = 1), South Africa (*n* = 1), Brazil (*n* = 1), Japan (*n* = 1), Kenya (*n* = 1), and Thailand (*n* = 1). The study participants included 522 healthcare policymakers or developers, nursing leaders with experiences in healthcare policy intervention, nursing association leaders, civic activists, and politicians who are nurses. They had extensive experiences in various healthcare policy interventions and political activities across central or local governments, the National Assembly, provincial assemblies, the Ministry of Health, public health agencies, nursing associations, civic groups, and political organizations (Supporting Table 4).

Qualitative statements from the studies used in the analysis included statements on nurses’ healthcare policy competence, political competence, political participation and policy intervention experiences, community nursing competence, and public health competence. The included studies used five phenomenological methods, four grounded theories, three case studies, 11 descriptive qualitative studies, and four mixed methods. Data collection consisted of 25 purposive samples and two voluntary samples. The interview methods used were semistructured individual in‐depth interviews, focus groups, face‐to‐face interviews, telephone interviews, and Delphi studies (Table 1).

**TABLE 1:** Characteristics of the included articles for analysis.

Author; country	Phenomenon of interest	Design/analysis	Setting/context/culture	Study’s participants	Data collection
Aarabi et al. [35]; Iran	Describing factors affecting the involvement of nurse leaders in policymaking for nursing	Exploratory‐qualitative study; conventional content analysis approach	Iran’s Board of Nursing, members of the Supreme Council of the Iranian Nursing organization	Eighteen nurse leaders who had key positions in policymaking for nursing	Purposive sampling; semistructured individual interviews; face‐to‐face interviews
Barry [36]; United States of America	Explaining the political socialization process of nurses who have taken on professional roles in government	Exploratory‐qualitative study; descriptive survey study	Positioning in congressional offices, regulatory agencies, or state legislatures, and willingness to participate	Thirty‐three nurses who had attained specialized roles in public policy development in the federal and state governments	Purposive sampling; semistructured/open‐ended in‐depth individual interviews; face‐to‐face and by telephone
Cheraghi et al. [37]; Iran	Elucidating Iranian nurses’ status in policymaking for nursing in the health system	Exploratory‐qualitative study; content analysis	Composed mostly of leaders who held senior policymaking positions in Iran’s Ministry of Health or the Iranian Nursing Organization	Twenty nurse leaders of management or policymakers with nursing policymaking	Purposive sampling; Semistructured individual interviews; face‐to‐face interviews
Chiu et al. [38]; Canada	Analysis of Professional Nursing Associations’ Participation in Policy Advocacy	Exploratory‐qualitative study; descriptive study	Regional, national, and international professional nursing associations, and executives from eight professional nursing associations	Staff leaders from eight professional nursing associations	Purposive sampling; semistructured individual interviews; face‐to‐face interviews
Deschaine and Schaffer [39]; United States of America	Recognizing factors that affect the ability of public health nurse leaders to influence health policy	Exploratory‐qualitative study; Longest’s (2002) public policymaking framework	A city or county public health agency	Eight PHN leaders representing rural, suburban, and urban areas in public health nursing	Purposive sampling; semistructured individual interviews; face‐to‐face interviews
Digaudio [40]; United States of America	Identifying the phenomenon of nurse policymaking as experienced by those in the trenches	Exploratory‐qualitative study; grounded theory	At least a baccalaureate degree in nursing	Twenty nurses who could identify and discuss a policymaking activity they experienced	Purposive sampling; semistructured individual interviews; open‐ended interviews
Ditlopo et al. [41]; South Africa	Dynamics, strengths, and weaknesses of nurse participation in four national workforce policies analyzed	Multiple descriptive case study: Thematic content analysis	Four provinces in South Africa: Gauteng, Eastern Cape, Free State, and the Western Cape	Twenty key informants and 73 frontline nurses in nursing policymaking	Purposive sampling; semistructured individual interviews; face‐to‐face interviews
Donovan et al. [42]; New Zealand	Investigating perceptions of nursing policy and political leadership	Exploratory‐qualitative study; constant comparative analysis	Fellows of the College of Nurses Aotearoa (NZ) Inc. who volunteered through a standard solicitation of the college	Eighteen nurse leaders from across the country	Volunteer sampling; semistructured interviews; face to face (16 nurses), telephone (two nurses)
Gebbie et al. [43]; United States	The role of American nurses in advancing health policy	Exploratory‐qualitative study	Provincial or national elected officials, national associations, and foundation representatives	Twenty‐seven U.S. nurse leaders involved in state and national policy	Purposive sampling; semistructured individual interviews
Hajizadeh et al. [18]; Iran	Exploring nurse managers’ attitudes and perceived benefits in the health policymaking process	Exploratory‐qualitative study; thematic analysis	Tabriz University of Medical Sciences, which has over 15 teaching hospitals in the northwest of Iran	Sixteen nurse managers, government officials, and faculty members	Purposive sampling; semistructured individual interviews; open‐ended in‐depth interviews
Hajizadeh et al. [12]; Iran	Exploring barriers and facilitators associated with nurse managers’ participation in the health policymaking process	Exploratory‐qualitative study; thematic analysis	Iranian nationality; nurses ≥ 25 years of age; a university degree; and worked in administrative positions for at least three years in a hospital	Sixteen nurse managers who are involved in the hospital or the government	Purposive sampling; semistructured individual interviews; open‐ended in‐depth interviews
Hahn [44]; United States of America	Experiences of national regulatory nurse leaders in compact policy agenda	Qualitative study followed a phenomenological hermeneutic methodology approach	Fifty U.S. state board of nursing members with nursing regulatory responsibilities	Twelve nurses who are members of the State Board of Nursing	Voluntary application sampling; focus group interviews (online)
Han [17]; South Korea	Exploring how South Korean nurses engage in civil society to reform healthcare policy	Exploratory‐qualitative study; phenomenology	Korean civic organizations’ activities for more than 5 years with a bachelor’s or higher degree	Seven Korean civic activist nurses who had led successful healthcare policy reforms	Purposive; snowball sampling; semistructured individual interviews; in‐depth interviews
Inayat et al. [19]; Canada	Exploring strategies to enhance nurses’ involvement in policymaking from the perspective of nurse leaders	Exploratory‐qualitative study; thematic analysis approach	In four cities (Islamabad, Lahore, Karachi, and Rawalpindi) in Pakistan	Eleven nurse leaders with at least one year of experience in policymaking	Purposive; sampling; semistructured individual interviews; in‐depth interviews
Kerschner and Cohen [45]; United States of America	Examining a particular aspect of health policy formation: The lived experience of individual legislative decision‐making	Exploratory‐qualitative study; phenomenology	House and Senate of the state legislative health and welfare committees	Four state legislators, all members of the House or Senate health and welfare committees	Purposive and voluntary sampling; semistructured individual interviews; in‐depth interviews
Melo and Santos [46]; Brazil	Identification of nursing managers’ concept of political participation in the Brazilian public health care system	Exploratory‐qualitative study; case study; thematic analysis	Qualified in full municipal management for at least two years, and having party‐political continuity	Nine managers or comanager nurses who have a position as a PHN with a leadership capacity	Purposive sampling; semistructured individual interviews; face‐to‐face interviews
Michibayashi et al. [20]; Japan	Identifying competencies of public health nurses working on local tobacco control	Exploratory‐qualitative study	Nine local governments with advanced tobacco control	Twelve expert PHNs in charge of tobacco control from nine local governments	Purposive sampling; semistructured individual interviews; in‐depth interviews
Moore [47]; United States of America	Developing essential factors for influencing and implementing sound policy decisions	Exploratory‐qualitative study; collective case study: critical theory and feminist theory	Nursing education executives from public, private, and for‐profit higher education institutions	Eleven nursing education executives who have been in the executive position	Purposive; sampling; semistructured individual interviews; in‐depth interviews
Richter et al. [48] Canada	Presentation of research findings on factors that hinder and facilitate nurses’ participation in policymaking	Mixed‐methods study: Individual interviews, focus groups, surveys and document analysis	Nursing departments, clinics, and medical managers from each institution, and senior nursing leaders	Fifty nurses (10 from Jamaica, 17 from Kenya, 12 from Uganda, and 12 from South Africa)	Purposive; sampling; semistructured individual interviews/in‐depth interviews and focus groups
Shariff [49]; Kenya	Exploring the extent of involvement and leadership attributes required of nurse leaders in health policy development	Mixed method: qualitative and quantitative research; qualitative analysis; Statistical analysis	Three East African countries of Kenya, Uganda, and Tanzania	Thirty‐seven national nurse leaders who have experience in health policy development and work at the Ministry of Health, etc.	Purposive sampling; three‐round Delphi: Round 1: open‐ended survey; Rounds 2 and 3: qualitative/quantitative data
Taylor [50]; United States of America	Eliciting insights from public policy leaders on ongoing advocacy initiatives that motivate nurses.	Descriptive web‐based survey design; social cognitive theory; content analysis	Twelve regional professional nursing organizations were designated as expert mentors	Twelve executive leadership and board committee members from their respective organizations	Purposive convenience sampling; initial web‐based electronic survey; semistructured web interview
Waddell et al. [51]; United States of America	Describing the experiences of nurse leaders working to influence policy	Mixed‐methods study: Delphi study/Focus group/Content analysis	Action coalition leadership team from a New England state	Twenty‐two nurse leaders who were members of a state action coalition	Purposive convenience sampling; Delphi study; focus group interviews; semistructured individual interviews
Warner [52]; United States of America	Understanding the art of political competence from the stories of six political nurse activists	Exploratory‐qualitative study; phenomenology	Having experience in appointed and elected office, organizational leadership, and federal healthcare reform activities	Six politically expert nurse activists	Purposive sampling; semistructured individual interviews; open‐ended in‐depth interviews
Wichaikhum et al. [53]; Thailand	Developing a strategic model for nurse policy development engagement in Thailand	Mixed method: qualitative and quantitative study: qualitative analysis; quantitative analysis; validity	Policy development at the organizational or national level, or working for nursing professional organizations with at least 10 years of experience	Fifteen nurse experts who have participated in policy development	Purposive sampling; three‐round Delphi method: open‐ended individual interviews
Williams [54]; United States of America	Understanding of advocacy concepts and practices aimed at developing public policy	Exploratory‐qualitative study: Grounded theory	Working in the states in various nursing/political advocacy roles; Virginia, Washington DC, Maryland	Ten nurses who were active in political advocacy as part of their nursing practice	Purposive sampling; semistructured interviews; open‐ended in‐depth interviews; face‐to face
Wilson [55]; United States of America	How the nursing profession influences the policymaking process	Exploratory‐qualitative study: Phenomenology	Substantive experience in high‐level or influential positions in organizations, institutions, or the government	Eight influential nursing leaders, and two non‐nurse healthcare leaders	Purposive sampling; semistructured individual interviews; in‐depth interviews
Wilson et al. [13]; Canada	Explaining why and how nurses became politically active and what they have achieved	Exploratory‐qualitative study: grounded theory	Political position, spokesperson, or leader of a political action group	Ten elected or politically active Canadian nurses	Purposive sampling; semistructured individual interviews; telephone interview

Researchers conducted a quality assessment using the Critical Appraisal Skills Programme [56] checklist, comprising ten items that evaluate the credibility, integrity, and trustworthiness of the individual studies used in the analysis. The CASP criteria assess the quality of studies and the internal validity of meta‐analyses and help reduce bias in individual studies. A higher score for meeting the question indicated that the individual study was more likely to have been conducted systematically. In this regard, Sandelowski and Barroso [57] recommend that the quality assessment of studies in qualitative meta‐synthesis should not be used solely as a selection criterion for individual studies but rather as a means to help understand them. Therefore, this study focused on identifying the strengths and weaknesses of each study using CASP and performed a more careful analysis of the synthesis and interpretation of results without excluding any studies. Researchers independently evaluated and compared individual studies and discussed disagreements until a consensus was reached. Table 2 lists the results of the CASP quality assessment of the included studies. All 27 articles met at least seven criteria, of which 22 (81.5%) met all criteria and 24 (88.9%) received ethical approval.

**TABLE 2:** Methodological quality appraisal according to the CASP criteria.

Selected studies	Q1	Q2	Q3	Q4	Q5	Q6	Q7	Q8	Q9	Q10
Aarabi et al. [35]	Y	Y	Y	Y	Y	Y	Y	Y	Y	Y
Barry [36]	Y	Y	Y	Y	Y	Y	N	Y	Y	Y
Cheraghi et al. [37]	Y	Y	Y	Y	Y	Y	Y	Y	Y	Y
Chiu et al. [38]	Y	Y	Y	Y	Y	U	Y	Y	Y	Y
Deschaine and Schaffer [39]	Y	Y	Y	Y	Y	Y	N	U	Y	Y
Digaudio [40]	Y	Y	Y	Y	Y	Y	Y	Y	Y	Y
Ditlopo et al. [41]	Y	Y	Y	Y	Y	Y	Y	Y	Y	Y
Donovan et al. [42]	Y	Y	Y	Y	Y	Y	Y	Y	Y	Y
Gebbie et al. [43]	Y	Y	Y	Y	Y	Y	N	U	Y	Y
Hajizadeh et al. [18]	Y	Y	Y	Y	Y	Y	Y	Y	Y	Y
Hajizadeh et al. [12]	Y	Y	Y	Y	Y	Y	Y	Y	Y	Y
Hahn [44]	Y	Y	Y	Y	Y	Y	Y	Y	Y	Y
Han [17]	Y	Y	Y	Y	Y	Y	Y	Y	Y	Y
Inayat et al. [19]	Y	Y	Y	Y	Y	Y	Y	Y	Y	Y
Kerschner and Cohen [45]	Y	Y	Y	Y	Y	Y	Y	Y	Y	Y
Melo and Santos [46]	Y	Y	Y	Y	Y	N	Y	U	N	Y
Michibayashi et al. [20]	Y	Y	Y	Y	Y	Y	Y	Y	Y	Y
Moore [47]	Y	Y	Y	Y	Y	Y	Y	Y	Y	Y
Richter et al. [48]	Y	Y	Y	Y	Y	Y	Y	Y	Y	Y
Shariff [49]	Y	Y	Y	Y	Y	Y	Y	Y	Y	Y
Taylor [50]	Y	Y	Y	Y	Y	Y	Y	Y	Y	Y
Waddell et al. [51]	Y	Y	Y	Y	Y	Y	Y	Y	Y	Y
Warner [52]	Y	Y	Y	Y	Y	Y	Y	Y	Y	Y
Wichaikhum et al. [53]	Y	Y	Y	Y	Y	Y	Y	Y	Y	Y
Williams [54]	Y	Y	Y	Y	Y	Y	Y	Y	Y	Y
Wilson [55]	Y	Y	Y	Y	Y	Y	Y	Y	Y	Y
Wilson et al. [13]	Y	Y	Y	Y	Y	Y	Y	Y	Y	Y

*Note:* Q: Question; Y: Yes; N: No; U: Unclear. Q1: Clear aims statement of the research; Q2: Appropriate qualitative methodology; Q3: Appropriate research design; Q4: Appropriate recruitment strategy appropriate to address the aim; Q5: Appropriate research method of data collection; Q6: Relationship considered between researchers and participants; Q7: Ethical issues considered; Q8: Sufficient rigorous data analysis; Q9: Clear statement of findings; Q10: Valuable research results.

### 2.4. Data Extraction and Meta‐Synthesis

Researchers extracted data, including research information, results, and quotations from the selected studies, using ATLAS.ti Version 24. ATLAS.ti is a qualitative data analysis software that supports more effective management, coding, classification, and analysis of unstructured data such as interviews or complex datasets. The software makes it easier to explore and understand the nature of the phenomena of interest. Researchers independently coded all statements from individual studies focused on the research objectives.

The qualitative meta‐synthesis was conducted in seven stages based on meta‐ethnography [25]. First, the purpose and reasons for using meta‐ethnography were explained at the beginning of this study. A systematic review was then conducted using databases and manual searches, and the results of the selected individual studies were analyzed using EndNote. After reading the selected individual studies several times, the characteristics and findings of the studies were extracted and organized on a data extraction sheet, and the primary data were identified. ATLAS.ti was then used to determine the relevance and concepts of the individual study results. The researchers independently coded the research participants’ translations of the “first‐order constructs” and analyzed the primary authors’ translations of the “second‐order constructs.” The researchers read the “first‐order constructs” and “second‐order constructs” intensively and repeatedly until the concepts aligned with the research objectives were clear and compared the results. Any disagreements between the researchers during the coding process were resolved through discussion. The researchers translated the coding results, compared the similarities and differences, and combined and classified similar themes and concepts. The relevance of each concept was then analyzed to derive “third‐order constructs,” which were abstracted into factors, themes, and subthemes that reflected the main themes of the study. During the analysis and synthesis processes, consensus was reached through continuous discussion to resolve the differences in interpretation between the two researchers. The synthesized results were checked for consistency with the original statements of the participants in the individual papers. The results were verified by nursing professors with extensive expertise in qualitative research and policy intervention. Finally, the results of the concept synthesis using meta‐ethnography were presented (Supporting Table 5), and the reliability of the qualitative evidence synthesis results was verified using the GRADE‐CERQual assessment [58] (Supporting Table 6). This study also complied with the eMERGe reporting criteria for meta‐ethnography (Supporting Table 7).

The researchers have diverse academic backgrounds, including studying nursing, medicine, and health sciences, as well as teaching nursing management and healthcare policy. They conducted qualitative research, including developing political competence concepts and measurement tools, policy analysis, and phenomenology. In addition, the researchers had experience serving as advisors to central and local governments on healthcare policy and working as directors of nursing organizations.

### 2.5. Synthesis Findings

**FIGURE 2:**
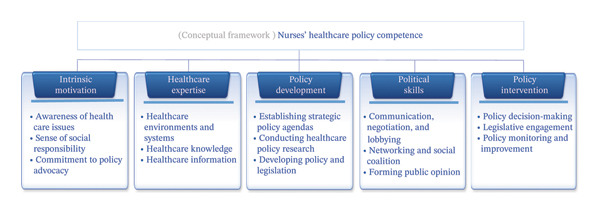
Findings of this study.

Three hundred and fifty‐seven findings on the nurses’ healthcare policy competence were derived from an analysis of quotations and individual study concepts. The findings were then synthesized into five factors, 15 themes, and 34 subthemes: intrinsic motivation (synthesized themes: awareness of healthcare issues, sense of social responsibility, commitment to policy advocacy) (healthcare expertise synthesized themes: healthcare environments and systems, healthcare knowledge, healthcare information); policy development (synthesized themes: establishing strategic policy agendas, conducting healthcare policy research, developing policy and legislation); political skills (synthesized themes: communication, negotiation, and lobbying, networking and social coalition, forming public opinion); and policy intervention (synthesized themes: policy decision‐making, legislative engagement, policy monitoring and improvement) (Table 3, Figure 2).

**TABLE 3:** Results of the synthesized findings and GRADE‐CERQual assessment.

Factors	Themes	Subthemes	References	Finding	Methodological limitation	Adequacy	Coherence	Relevance	CERQual assessment of confidence in the evidence
Intrinsic motivation	Awareness of healthcare issues	Perception of healthcare environment issues	[13, 17, 18, 20, 36, 37, 40, 42, 46, 48–50, 52–55]	8	Minor methodological limitations: 2/16 study with a lack of consideration of ethical issues	No concerns about the coherence of data	No concerns about adequacy of data (16 studies)	No concerns about the relevance of data (16 studies)	High confidence: The two studies of moderate quality, with minor methodological limitations, high coherence, high relevance, and no concerns about data adequacy
		Recognition of healthcare policy problems	[17, 20, 40, 42, 48, 50, 52, 54]	6					
	Sense of social responsibility	Recognizing political responsibility as a nursing professional	[13, 17, 19, 20, 35, 40, 41, 43, 46, 50–54]	5	Minor methodological limitations: 1/17 study with a lack of consideration of ethical issues and 1/17 study with unclear evidence of reflexivity	No concerns about the coherence of data	No concerns about adequacy of data (17 studies)	No concerns about the relevance of data (17 studies)	High confidence: The one study of moderate quality, with minor methodological limitations, high coherence, high relevance, and no concerns about data adequacy
		Interest in politics or healthcare policy	[12, 13, 17, 18, 40, 41, 50, 52–54]	4					
	Commitment to policy advocacy	Sense of nursing values based on nursing identity	[12, 13, 17, 20, 40–44, 46, 47, 50, 52–54]	9	Minor methodological limitations: 2/17 study with a lack of consideration of ethical issues and 2/17 study with unclear evidence of reflexivity	No concerns about the coherence of data	No concerns about adequacy of data (17 studies)	No concerns about the relevance of data (17 studies)	High confidence: The two studies of moderate quality, with minor methodological limitations, high coherence, high relevance, and no concerns about data adequacy
		Willingness and persistence to drive policy change	[17, 18, 20, 36, 40–44, 47, 49–54]	13					

Healthcare expertise	Healthcare environments and systems	Identifying healthcare environments	[17, 20, 36, 40, 42, 46, 50, 52–55]	3	Minor methodological limitations: 1/13 study with a lack of consideration of ethical issues and 1/13 study with unclear evidence of reflexivity	No concerns about the coherence of data	No concerns about adequacy of data (13 studies)	No concerns about the relevance of data (13 studies)	High confidence: The one study of moderate quality, with minor methodological limitations, high coherence, high relevance, and no concerns about data adequacy
		Understanding healthcare systems and delivery models	[17, 20, 36, 40–42, 46, 50, 52–55]	2					
	Healthcare knowledge	Understanding the healthcare policymaking process	[12, 13, 17, 19, 20, 36, 39, 40, 42, 43, 50, 52–55]	13	Moderate methodological limitations: 3/24 study with a lack of consideration of ethical issues and 4/24 study with unclear evidence of reflexivity	No concerns about the coherence of data	No concerns about adequacy of data (24 studies)	No concerns about the relevance of data (24 studies)	Moderate confidence: the four studies of moderate quality, with minor methodological limitations, high coherence, high relevance, and no concerns about data adequacy
		Understanding the complex nature of the political landscape and resource constraints	[13, 17, 36, 39, 40, 42, 46, 47, 49–51, 53, 54]	10					
	Healthcare information	Gaining healthcare issues or problems	[17, 18, 35, 37, 39, 42, 43, 45, 46, 50, 52–55]	4	Moderate methodological limitations: 3/19 study with a lack of consideration of ethical issues and 4/19 study with unclear evidence of reflexivity	No concerns about the coherence of data	No concerns about adequacy of data (19 studies)	No concerns about the relevance of data (19 studies)	Moderate confidence: the four studies of moderate quality, with minor methodological limitations, high coherence, high relevance, and no concerns about data adequacy
		Collecting healthcare information that influences the policy process	[12, 17, 20, 35–40, 42, 45, 46, 49, 50, 52–54]	6					

Policy development	Establishing strategic policy agendas	Setting the healthcare policy agenda and plan	[17, 20, 35–37, 39, 40, 42–44, 46, 47, 49, 50, 52–54]	19	Minor methodological limitations: 3/18 study with a lack of consideration of ethical issues and 3/18 study with unclear evidence of reflexivity	No concerns about the coherence of data	No concerns about adequacy of data (18 studies)	No concerns about the relevance of data (18 studies)	Moderate confidence: The three studies of moderate quality, with minor methodological limitations, high coherence, high relevance, and no concerns about data adequacy
		Considering the equity, efficiency, and feasibility of healthcare policy	[20, 35, 40, 45, 54]	7					
	Conducting healthcare policy research	Analyzing and interpreting datasets based on critical thinking	[12, 17–20, 36, 40, 42, 43, 45, 46, 52–54]	17	Moderate methodological limitations: 3/19 study with a lack of consideration of ethical issues and 3/19 study with unclear evidence of reflexivity	No concerns about the coherence of data	No concerns about adequacy of data (19 studies)	No concerns about the relevance of data (19 studies)	Moderate confidence: The three studies of moderate quality, with minor methodological limitations, high coherence, high relevance, and no concerns about data adequacy
		Generating research evidence to influence health policy	[12, 17, 20, 39, 40, 42, 43, 47, 50, 53, 54]	6					
	Developing policy and legislation	Developing healthcare policy and legislation	[12, 17, 18, 20, 36, 39, 40, 42–44, 47, 49, 50, 52–55]	9	Moderate methodological limitations: 3/18 study with a lack of consideration of ethical issues and 3/18 study with unclear evidence of reflexivity	No concerns about the coherence of data	No concerns about adequacy of data (18 studies)	No concerns about the relevance of data (18 studies)	Moderate confidence: The three studies of moderate quality, with moderate methodological limitations, high coherence, high relevance, and great concerns about data adequacy
		Disseminating evidence‐based developed policies	[17, 20, 40, 43, 45, 46, 50, 52–54]	6					

Political skills	Communication, negotiation, and lobbying	Clear articulation of the political perspective using effective listening, speaking, or writing	[12, 17, 20, 36, 37, 39, 42–47, 49–54]	23	Moderate methodological limitations: 3/21 study with a lack of consideration of ethical issues and 3/21 study with unclear evidence of reflexivity	No concerns about the coherence of data	No concerns about adequacy of data (21 studies)	No concerns about the relevance of data (21 studies)	Moderate confidence: the three studies of moderate quality, with moderate methodological limitations, high coherence, high relevance, and no concerns about data adequacy
		Persuasive communication with a variety of stakeholders or policymakers	[17, 20, 40, 42, 43, 49, 50, 52–54]	7					
		Negotiation skills to resolve conflicts and compromise	[12, 17, 20, 40, 42–45, 47, 49, 50, 52–55]	12					
		Political lobbying through establishing effective channels with policymakers	[17, 38–40, 42–44, 50, 52, 54, 55]	5					
	Networking and social coalition	Participating in nursing representative organizations and nongovernmental organizations	[13, 17–20, 36, 38, 40, 42–44, 49, 50, 52–55]	15	Moderate methodological limitations: 3/22 study with a lack of consideration of ethical issues and 4/22 study with unclear evidence of reflexivity	No concerns about the coherence of data	No concerns about adequacy of data (22 studies)	No concerns about the relevance of data (22 studies)	Moderate confidence: the four studies of moderate quality, with moderate methodological limitations, high coherence, high relevance, and no concerns about data adequacy
		Collaboration with professionals, various stakeholders, politicians, and civic groups	[17, 19, 20, 36, 40, 42–44, 46, 47, 49, 50, 52–54]	18					
		Developing network platforms with professionals, civic groups, politicians, and legislators	[12, 17, 18, 20, 38–40, 42–44, 46, 47, 49, 50, 52–54]	14					
		Obtaining political or economic support, resources, and information through networking	[19, 20, 40, 43, 47, 54]	7					
		Collective actions and a unified voice through social coalition	[13, 17, 19, 35, 36, 39, 40, 42, 47, 50, 52–55]	9					
	Forming public opinion	Using various media to promote policies and rally public support	[12, 13, 17, 19, 20, 38, 39, 43, 49, 50, 52–55]	23	Minor methodological limitations: 2/17 study with a lack of consideration of ethical issues and 2/17 study with unclear evidence of reflexivity	No concerns about the coherence of data	No concerns about adequacy of data (17 studies)	No concerns about the relevance of data (17 studies)	High confidence: the three studies of moderate quality, with moderate methodological limitations, high coherence, high relevance, and no concerns about data adequacy
		Operating conferences, policy workshops, or forums to share health issues and influence policy	[13, 17, 19, 43, 47–50, 52–54]	8					

Policy intervention	Policy decision‐making	Policy engagement in governmental committees or health departments and policy groups	[12, 13, 17–20, 36–41, 43–46, 49, 50, 52–55]	34	Moderate methodological limitations: 3/24 study with a lack of consideration of ethical issues and 4/24 study with unclear evidence of reflexivity	No concerns about the coherence of data	No concerns about adequacy of data (24 studies)	No concerns about the relevance of data (24 studies)	Moderate confidence: the four studies of moderate quality, with moderate methodological limitations, high coherence, high relevance, and no concerns about data adequacy
		Creating more opportunities through successful policy decision‐making experiences	[17, 20, 36, 38, 40, 42, 50, 52–54]	6					
	Legislative engagement	Engagement with the government and the legislation process	[13, 17, 18, 20, 36, 37, 39, 40, 44, 46, 48–50, 52–55]	13	Minor methodological limitations: 2/17 study with a lack of consideration of ethical issues and 1/17 study with unclear evidence of reflexivity	No concerns about the coherence of data	No concerns about adequacy of data (17 studies)	No concerns about the relevance of data (17 studies)	High confidence: the two studies of moderate quality, with moderate methodological limitations, high coherence, high relevance, and high concerns about data adequacy
	Policy monitoring and improvement	Monitoring the policy implementation process	[17, 20, 48, 50, 54]	11	No methodological limitations: 5/5 studies with clear evidence of reflexivity	No concerns about the coherence of data	No concerns about adequacy of data (five studies)	No concerns about the relevance of data (five studies)	High confidence: no methodological limitations, high coherence, high relevance, and no concerns about data adequacy
		Evaluating and improving the quality of healthcare policy implementation	[17, 20, 50, 52, 55]	5					

### 2.6. Factor 1: Intrinsic Motivation

Intrinsic motivation, a factor of nurses’ healthcare policy competence, was inductively synthesized into three themes and six subthemes: awareness of healthcare issues (synthesized subthemes: perception of healthcare environment issues; recognition of healthcare policy problems), sense of social responsibility (synthesized subthemes: recognizing political responsibility as a nursing professional; interest in politics or healthcare policy), and commitment to policy advocacy (synthesized subthemes: sense of nursing values based on nursing identity; willingness and persistence to drive policy change).

Most nurse political activists emphasized the importance of motivation for engaging in healthcare policy interventions and stated “intrinsic motivation” as one of the healthcare policy competencies required of nurses. “Intrinsic motivation” competence included “awareness of healthcare issues” [13, 17, 18, 20, 36, 37, 40, 42, 46, 48–50, 52–55], which encompasses the perception of healthcare environment issues [13, 17, 18, 20, 36, 37, 40, 42, 46, 48–50, 52–55] and healthcare policy problems [17, 20, 40, 42, 48, 50, 52, 54].

Intrinsic motivation also embedded a “sense of social responsibility” [12, 13, 17–20, 35, 40, 41, 43, 46, 50–54], which encompasses recognizing political responsibility as a nursing professional [13, 17, 19, 20, 35, 40, 41, 43, 46, 50–54] and the interest in politics or healthcare policy [12, 13, 17, 18, 40, 41, 50, 52–54].

In addition, “Intrinsic motivation” included “commitment to policy advocacy” [12, 13, 17, 18, 20, 36, 40–44, 46, 47, 50, 52–54], which implies a sense of nursing values based on nursing identity [12, 13, 17, 20, 40–44, 46, 47, 50, 52–54] and willingness and persistence to drive policy change [17, 18, 20, 36, 40–44, 47, 49–54].

### 2.7. Factor 2: Healthcare Expertise

Healthcare expertise, a factor of nurses’ healthcare policy competence, was synthesized into three themes and six subthemes: healthcare environments and systems (subthemes: identifying healthcare environments and understanding healthcare systems and delivery models), healthcare knowledge (subthemes: understanding the healthcare policymaking process; and understanding the complex nature of the political landscape and resource constraints), and healthcare information (subthemes: gaining healthcare issues or problems; collecting healthcare information that influences the policy process).

Most of the participants stated that “healthcare expertise” is essential competence for nurses to engage in healthcare policy. “Healthcare expertise” included “healthcare environments and systems” [17, 20, 36, 40–42, 46, 50, 52–55], which implies identifying healthcare environments [17, 20, 36, 40, 42, 46, 50, 52–55] and understanding healthcare systems and delivery models [17, 20, 36, 40–42, 46, 50, 52–55].

Healthcare expertise also implied “healthcare knowledge” [12, 13, 17, 19, 20, 36, 38–40, 42, 43, 46, 47, 49–55], which encompasses understanding the healthcare policymaking process [12, 13, 17, 19, 20, 36, 39, 40, 42, 43, 50, 52–55] and understanding the complex nature of the political landscape and resourceful constraints [13, 17, 36, 39, 40, 42, 46, 47, 49–51, 53, 54]. Furthermore, “healthcare expertise” included “healthcare information” [12, 17, 18, 20, 35–40, 42, 43, 45, 46, 49, 50, 52–55], which implies gaining healthcare issues or problems [17, 18, 35, 37, 39, 42, 43, 45, 46, 50, 52–55] and collecting healthcare information that influences the policy process [12, 17, 20, 35–40, 42, 45, 46, 49, 50, 52–54].

### 2.8. Factor 3: Policy Development

Policy development, a factor of nurses’ healthcare policy competence, was synthesized into three themes and six subthemes: establishing strategic policy agendas (subthemes: setting the healthcare policy agenda and plan; considering the equity, efficiency, and feasibility of healthcare policy), conducting healthcare policy research (subthemes: analyzing and interpreting datasets based on critical thinking; generating research evidence to influence health policy), and developing policy and legislation (subthemes: developing healthcare policy and legislation; disseminating evidence‐based developed policies).

Nurse political activists reported that strengthening “policy development” ability was essential. Policy development included “establishing strategic policy agendas” [17, 20, 35–37, 39, 40, 42–47, 49, 50, 52–54], which encompasses setting the healthcare policy agenda and plan [17, 20, 35–37, 39, 40, 42–44, 46, 47, 49, 50, 52–54] and considering the equity, efficiency, and feasibility of healthcare policy [20, 35, 40, 45, 54].

Policy development also embedded a “conducting healthcare policy research” [12, 17–20, 36, 39, 40, 42, 43, 45–47, 50, 52–54], which embraces analyzing and interpreting datasets based on critical thinking [12, 17–20, 36, 40, 42, 43, 45, 46, 52–54] and generating research evidence to influence health policy [12, 17, 20, 39, 40, 42, 43, 47, 50, 53, 54].

In addition, “policy development” included “developing policy and legislation” [12, 17, 18, 20, 36, 39, 40, 42–44, 46, 47, 49, 50, 52–55], which means developing healthcare policy and legislation [12, 17, 18, 20, 36, 39, 40, 42–44, 47, 49, 50, 52–55] and disseminating evidence‐based developed policies [17, 20, 40, 43, 45, 46, 50, 52–54].

### 2.9. Factor 4: Political Skills

Political skills, a factor of nurses’ healthcare policy competence, was synthesized into three themes and 11 subthemes: communication, negotiation, and lobbying (subthemes: clear articulation of the political perspective using effective listening, speaking, or writing; persuasive communication with a variety of stakeholders or policymakers; negotiation skills to resolve conflicts and compromise; and political lobbying through establishing effective channels with policymakers), networking and social coalition (subthemes: participating in nursing representative organizations and nongovernmental organizations; and collaboration with professionals, various stakeholders, politicians, and civic groups; developing network platforms with professionals, civic groups, politicians, and legislators; obtaining political or economic support, resources, and information through networking; collective actions and a unified voice through social coalition), forming public opinion (subthemes: using various media to promote policies and rallying public support; operating conferences, policy workshops, or forums to share health issues and influence policy).

Most participants emphasized the importance of “political skills” to facilitate nurses’ intervention in healthcare policy. Political skills included “communication, negotiation, and lobbying” [12, 17, 20, 36, 37, 39, 40, 42–45, 47, 49–55], which encompassed the clear articulation of political perspective using effective listening, speaking, or writing [12, 17, 20, 36, 37, 39, 42–47, 49–54], persuasive communication with a variety of stakeholders and policymakers [17, 20, 40, 42, 43, 49, 50, 52–54], negotiation skills to resolve conflicts and compromise [12, 17, 20, 40, 42–45, 47, 49, 50, 52–55], and political lobbying through establishing effective channels with policymakers [17, 38–40, 42–44, 50, 52, 54, 55].

Political skills also embedded a “networking and social coalition” [12, 13, 17–20, 35, 36, 39, 40, 42–44, 46, 47, 49, 50, 52–55], which embraces participating in nursing representative organizations and nongovernmental organizations [13, 17–20, 36, 40, 42–44, 49, 50, 52–55], collaboration with professionals, various stakeholders, politicians, and civic groups [17, 19, 20, 36, 40, 42–44, 46, 47, 49, 50, 52–54], developing network platforms with professionals, civic groups, politicians, and legislators [12, 17, 18, 20, 38–40, 42–44, 46, 47, 49, 50, 52–54], obtaining political or economic support, resources, and information through networking [19, 20, 40, 43, 47, 54], and collective actions and a unified voice through social coalition [13, 17, 19, 35, 36, 39, 40, 42, 47, 50, 52–55].

In addition, “political skills” included “forming public opinion” [12, 13, 17, 19, 20, 39, 43, 47, 49, 50, 52–55], which implies using various media to promote policies, rally public support [12, 13, 17, 19, 20, 38, 39, 43, 49, 50, 52–55] and operate conferences, policy workshops, or forums to share health issues and influence policy [13, 17, 19, 43, 47–50, 52–54].

### 2.10. Factor 5: Policy Intervention

Policy intervention, a factor of nurses’ healthcare policy competence, was synthesized into three themes and five subthemes: policy decision‐making (subthemes: policy engagement in governmental committees or health departments and policy groups; creating more opportunities through successful policy decision‐making experiences), legislative engagement (subthemes: engagement with the government and the legislation process), and policy monitoring and improvement (subthemes: monitoring the policy implementation process; evaluating and improving the quality of healthcare policy implementation).

Nurse political activists mentioned that nurses’ healthcare policy competence requires “policy intervention” ability. “Policy intervention” competence included “policy decision‐making” [12, 13, 17–20, 36, 37, 39–46, 49, 50, 52–55], which encompasses policy engagement in governmental committees or health departments and policy groups [12, 13, 17–20, 36–41, 43–46, 49, 50, 52–55] as well as the development of additional opportunities through successful policy decision‐making experiences [17, 20, 36, 38, 40, 42, 50, 52–54].

Policy intervention also embedded a “legislative engagement,” which embraces engagement with government and the legislation process [13, 17, 18, 20, 36, 37, 39, 40, 44, 46, 48–50, 52–55].

Furthermore, “policy intervention” includes “policy monitoring and improvement” [17, 20, 50, 52, 54, 56], which means monitoring the policy implementation process [17, 20, 48, 50, 54] and evaluating and improving the quality of healthcare policy implementation [17, 20, 50, 52, 56].

## 3. Discussion

This study conducted a systematic review and meta‐synthesis of previous studies to analyze the concept of nurses’ healthcare policy competence. Five factors were derived: intrinsic motivation, healthcare expertise, policy development, political skills, and policy intervention.

First, the “intrinsic motivation” factor of nurses’ competence in healthcare policy [12, 13, 17–20, 35–37, 40–44, 46–55] implies the nurses’ ability to commit to healthcare policy reform continuously based on an awareness of healthcare issues and problems, social responsibility for improving nurses’ rights, and advocacy for public health as nursing professionals. Cohen et al. [59] proposed four stages of nursing professional policy intervention or political participation: buy‐in, sell‐interest, political sophistication, and leading the way. Among these, the concept related to the “intrinsic motivation” factor is “buy‐in” as the first phase, which emphasizes the recognition of political activism as a means to achieve the goals of the nursing professional. Through this, the nurses’ intervention in healthcare policy is activated, and when legislating or establishing public health polices, the perspective of nursing professionals could be reflected in the distribution of healthcare resources. The “intrinsic motivation” factor is related to political efficacy in political science, which is the most important and widely used concept in political theory and refers to the perception that individuals can play a role in bringing about political and social change. Therefore, a higher level of political efficacy, an influential variable in explaining policy intervention and political participation, is associated with a higher level of involvement [60]. In addition, “intrinsic motivation” is a fundamental cognitive psychological factor that affects the other four factors of nurses’ healthcare policy competence.

The “healthcare expertise” factor of nurses’ healthcare policy competence [12, 13, 17–20, 35–43, 45–47, 49–55] includes understanding healthcare environments and systems, the policymaking process, and the complex nature of the political landscape and resource constraints, as well as gaining knowledge of healthcare issues or problems and collecting healthcare information that influences the policy process. Healthcare expertise is linked to the value of publicness [61] and serves as a tool for policy intervention and legislative intent [61]. Therefore, healthcare expertise is essential when determining methods and strategies to reform problematic policies or laws [47]. Han & Kim’s [11] systematic review and meta‐synthesis study on the barriers and facilitators of nurses “political participation or healthcare policy intervention, many political nursing activists mentioned that the most significant barrier to nurses” policy intervention is the lack of political knowledge and information because of the insufficient nursing education related to politics and healthcare policy. Therefore, enhancing nurses’ healthcare policy competence through formal education or program development is essential for increasing their healthcare expertise.

The “policy development” factor [12, 17–20, 35–37, 39, 40, 42–47, 49, 50, 52–54] refers to the ability to establish a strategic policy agenda and develop and disseminate policy and legislative alternatives by identifying healthcare issues and policy problems through analysis and research. Policy development is placing healthcare issues on the agenda of policymakers, preparing resolutions, and determining necessary resources [62]. It is an essential ability that must be possessed before healthcare policy intervention. In this regard, Longest’s model [10] in political science is a theoretical framework for public policymaking, which consists of the following: defining the problem to be solved, setting the policy agenda, participating in policy development, and converting problem solvability into action policy, which is consistent with the policy development ability in this study.

The political skills factor of nurses’ competence in healthcare policy [12, 13, 17–20, 35–55] implies the following attributes: communication, negotiation, and lobbying, networking and social coalition, and forming public opinion. Politics is related to the official systems of the government, the public sphere, and policies and is a social activity that expresses diverse opinions from interests [63]. In addition, politics is related to the distribution of power and resources [4]. Thus, it means making collective decisions regarding competition or conflict between interest groups, such as compromise, persuasion, adjustment, and negotiation [63, 64]. In particular, an important attribute of political skills is joining a nursing representative organization and actively participating in political activities, building networks and social alliances with various healthcare professionals, civic groups, politicians, and members of the National Assembly, and collectively exercising political influence [13, 54, 55]. In addition, forming public opinion through mass media, debates, press statements, and rallies is an essential political skill [65].

The “policy intervention” factor [12, 13, 17–20, 36–46, 48–50, 52–55] is the ability to influence the implementation of healthcare policy alternatives by intervening in the policymaking or legislative process to reflect nursing perspectives and by evaluating and improving implemented reform policies to ensure they are not distorted. Policy intervention ability corresponds to the outcome attribute of nurses’ healthcare policy competence. It is a competence that can be achieved only when the four‐factor abilities operate dynamically and organically. In other words, the activation of policy intervention competency is possible when the level of four‐factor abilities is sufficiently high to develop policy and effectively use various political skills by establishing healthcare expertise based on the intrinsic motivation that underlies healthcare policy competence. Healthcare policy reform should not be carried out hastily because limited resources must be distributed in consideration of efficiency, equity, and sustainability, and it is necessary to evaluate and improve performance during the policy implementation process regularly [13, 66]. Therefore, the appropriate division of roles between the public and private sectors, the democratic nature of procedures, and the public nature and marketability of healthcare must be fully considered when applying policy interventions [9, 10].

This study comprehensively analyzed articles on nurses’ healthcare policy competence, public health nursing competence, community health nursing competence, and political participation experience competence related to this study to minimize the omission of research results. Researchers with extensive experience in qualitative research and policy intervention conducted this study through sufficient discussion and agreement throughout the research process to secure the validity of the study. The strength of this study lies in its ability to integrate research from multiple countries to deeply reconstruct the essential meaning of nurses’ competence in healthcare policy and provide a foundation for establishing a theoretical model. The conceptual framework for nurses’ competence in healthcare policy identified in this study can be applied to policies and nursing education programs aimed at strengthening the competence of nursing professionals within representative nursing organizations. Furthermore, nurse leaders and nurses in clinical or community nursing practice can use this conceptual framework to demonstrate public health leadership. Through this, the strengthened healthcare policy competence of nursing professionals—the largest group of healthcare professionals—can facilitate national health policy interventions, thereby promoting evidence‐based public health rights, improved nursing work environments, and the equity and efficiency of public health policies. The data analyzed in this study were limited to 522 nurse political activists in 10 countries, so the research results cannot be generalized. In addition, the personal backgrounds of the participants and the healthcare environments, systems, and healthcare policies of the countries to which the participants included in this study belong differ according to country, so the risk of selection bias cannot be excluded. Lastly, this study analyzed the qualitative data stated by the participants in published papers rather than raw data provided by individual studies because of the restrictions in obtaining data.

## 4. Conclusions

This study conducted a systematic review and qualitative meta‐synthesis of previous research results to analyze the healthcare policy competence of nurses comprehensively. As a result, nurses’ competence in healthcare policy was synthesized into five factors (intrinsic motivation, healthcare expertise, policy development, political skills, and policy intervention), and 15 themes and 34 subthemes were derived as subattributes. This study provides a conceptual framework for nurses’ competence in healthcare policy and for developing political or policy education programs to strengthen this competence. This framework could also serve as primary data for developing a theory to enhance the future healthcare policy competence of nurses.

## Funding

This research was funded by the Academic Research Fund of Hoseo University (2025‐0162‐01).

## Conflicts of Interest

The authors declare no conflicts of interest.

## Supporting Information

Supporting information includes seven files that provide further information about the selection criteria for the study (PICOTS‐SD), database search strategies, excluded articles based on the full‐text review, selected articles for analysis, the results of synthesis findings, the GRADE‐CERQual assessment, and the eMERGE reporting result (Supporting Information).

## Supporting information


**Supporting Information** Additional supporting information can be found online in the Supporting Information section.

## Data Availability

The data that support the findings of this study are available in the Supporting Information of this article.
