# Supplementary material for: Exploring the Concept of Nurses’ Healthcare Policy Competence: A Systematic Review and Qualitative Meta‐Synthesis
Source: J Nurs Manag. 2026 Mar 20;2026:9182331. doi: 10.1155/jonm/9182331 (PMC13140810; doi:10.1155/jonm/9182331)
Supplement: Supplementary file 1 — Supporting Information Additional supporting information can be found online in the Supporting Information section. [file JONM-2026-9182331-s001.docx]

**Supplementary materials.**

**Supplementary table 1.** **Selection criteria for the study (PICOTS-SD).**

The participants in the individual studies included in this study were nurses, and the phenomena of interest were previous studies on nurses’ healthcare policy competence. The search was conducted from September 5, 2023, to March 30, 2024, and qualitative studies and doctoral dissertations published in English in journals by database until December 31, 2023, were included. The scope of qualitative studies included ethnography, grounded theory, phenomenology, and qualitative descriptive studies that described participants’ statements. Exclusion criteria included non-nurses, other healthcare professionals, nursing students, quantitative studies, perspectives, and commentaries.

| Classification | Inclusion criteria | Exclusion criteria |
| --- | --- | --- |
| Participants | ▪Nurses  ▪Registered nurses  ▪Licensed nurses  ▪Nursing profession  ▪Nursing professional  ▪Midwifery  ▪Midwives | ▪Non-nurse occupations; Nursing personnel or staff not including nursing professionals, healthcare personnel, healthcare profession, and other occupation |
| Interventions/Exposure | ▪Nurses’ healthcare policy competence  ▪Nurses’ political efficacy, political knowledge, policy development, political skills,  and policy intervention | ▪Non-nurses’ healthcare policy competence  ▪Nurses’ practice competence  ▪Organizational or national policy competence |
| Comparisons/Control | ▪Not applicable | ▪Not applicable |
| Outcomes: Phenomenon of Interest | ▪Exploring the concept of nurses’ healthcare policy competence | ▪Nurses’ practice competence in the hospital  ▪Community health nurses’ administrative competence in public health centers  ▪Public health nurses’ administrative competence in public health centers  ▪Public health nurses’ health promotion competence in public health centers  ▪Results of healthcare policy analysis  ▪Results of general national policy analysis |
| Time | ▪Articles published from the first publication to December 31, 2023, by each database of search | ▪Articles published after December 31, 2023 |
| Setting | ▪National/local healthcare policy or advocacy or politics | ▪Organizational policy or politics  ▪Non-English papers |
| Study Design | ▪Empirical research  ▪Qualitative study  ▪Descriptive study  ▪Exploratory study  ▪Empirical research  ▪Grounded theory  ▪Phenomenology  ▪Ethnography  ▪Case Study  ▪Focus group interviews  ▪In-depth interviews  ▪Semi-structured interviews  ▪Unstructured interviews  ▪Face-to-face interview  ▪Fieldwork interview | ▪Quantitative studies  ▪Mixed methods research with absent or uncertain qualitative data  ▪Not a primary study: Interview, conference material, commentary, perspective, review, letter |

**Supplementary table 2. Database search strategies.**

The databases used in this study were Medline, Embase, PubMed, CINAHL, Web of Science, and Scopus based on the COre search electronic databases of COSI (COre Standard, Ideal) by the National Library of Medicine. In addition, a manual search was conducted via Google Scholar for additional searches. The search strategy was Nurse* OR Licensed nurse* OR Registered nurse* OR Nursing professional OR Nursing profession OR midwives OR midwifery OR midwife AND Politics OR Political OR Policy OR Policies OR Advocac* AND Legislat* OR Public health OR Advocac* AND Competenc* OR Experience* OR Capacit* OR Capabilit* OR Leadership OR abilit* OR Socialization OR Socialisation AND Qualitative* OR Interview* OR Empirical OR Fieldwork OR Field work OR Focus group* OR Unstructured OR In-depth OR Semi-structured OR Semistructured OR Indepth OR Face-to-face OR Phenomenolog* OR Grounded theory OR Ethnograph*.

| **1. PubMed (1908 – 31 December 2023)** | | |
| --- | --- | --- |
| #1 | “Nurse*”[mesh] OR “Licensed nurse*”[tiab] OR “Registered nurse*”[tiab] OR “midwives”[tiab] OR “midwifery”[tiab] OR “midwife”[tiab] OR “Nursing profession”[tiab] OR “Nursing professional”[tiab] | 356,584 |
| #2 | “Politics”[tiab] OR “Political”[tiab] “Policy”[tiab] OR “Policies”[tiab] OR “Legislat*”[tiab] OR “Public health”[tiab] OR “Advocac*”[tiab] | 582,946 |
| #3 | “Competenc*”[tiab] OR “Experience*”[tiab] OR “Capacit*”[tiab] “Capabilit*” [tiab] OR “Leadership”[tiab] OR “abilit*”[tiab] OR “Socialization”[tiab] OR “Socialisation”[tiab] | 1,258,226 |
| #4 | “Qualitative*”[tiab] OR “Interview*”[tiab] OR “Empirical”[tiab] OR “Fieldwork”[tiab] OR “Field work”[tiab] OR “Focus group*”[tiab] OR “Unstructured”[tiab] OR “In-depth”[tiab] OR “Semi-structured”[tiab] OR “Semistructured”[tiab] OR “Indepth”[tiab] OR “Face-to-face”[tiab] OR “Phenomenolog*”[tiab] OR “Grounded theory”[tiab] OR “Ethnograph*”[tiab] | 1,045,171 |
| #5 | #1 AND #2 AND #3 AND #4 | **677** |
| **2. EBSCOhost CINAHL (1937 – 31 December 2023)** | | |
| S1 | TI “Nurse*” OR AB “Nurse*” OR TI “Registered nurse*” OR AB “Registered nurse*” OR TI “Licensed nurse*” OR AB “Licensed nurse*” OR TI “Nursing professional” OR AB “Nursing professional” OR TI “Nursing profession” OR AB “Nursing profession” OR TI “midwifery” OR AB “midwifery” OR TI “midwife” OR AB “midwife” OR TI “midwives” OR AB “midwives” | 396,756 |
| S2 | TI “Policy” OR AB “Policy” OR TI “Policies” OR AB “Policies” OR TI “Politics” OR AB “Politics” OR TI “Political” OR AB “Political” OR TI “Legislat*” OR AB “Legislat*” OR TI “Public health” OR AB “Public health” OR TI “Advocac*” OR AB “Advocac*” | 323,046 |
| S3 | TI “Competenc*” OR AB “Competenc*” OR TI “Experience*” OR AB “Experience*” OR TI “Capacit*” OR AB “Capacit*” OR TI “Capabilit*” OR AB “capabilit*” OR TI “Leadership” OR AB “Leadership” OR TI “abilit*” OR AB “abilit*” OR TI “Socialization” OR AB “Socialization” OR TI “Socialisation” OR AB “Socialisation” | 826,689 |
| S4 | TI “Empirical research” OR AB “Empirical research” OR TI “Focus group*”OR AB “Focus group*” OR TI “Semi-structured” OR AB “Semi-structured” OR TI “Semistructured” OR AB “Semistructured” OR TI “Unstructured” OR AB “Unstructured” OR TI “In-depth” OR AB “In-depth” OR TI “Indepth” OR AB “Indepth” OR TI “Face-to-face” OR AB “Face-to-face” OR TI “Grounded theory” OR AB “Grounded theory” OR TI “Phenomenolog*” OR AB “Phenomenolog*” OR TI “Ethnograph*” OR AB “Ethnograph*” OR TI “Field work” OR AB “Field work” | 188,442 |
| S5 | S1 AND S2 AND S3 AND S4 | **2,817** |
| **3. EBSCOhost Medline (1946 – 31 December 2023)** | | |
| S1 | TI “Nurse*” OR AB “Nurse*” OR TI “Registered nurse*” OR AB “Registered nurse*” OR TI “Licensed nurse*” OR AB “Licensed nurse*” OR TI “Nursing professional” OR AB “Nursing professional” OR TI “Nursing profession” OR AB “Nursing profession” OR TI “midwifery” OR AB “midwifery” OR TI “midwife” OR AB “midwife” OR TI “midwives” OR AB “midwives” | 346,260 |
| S2 | TI “Policy” OR AB “Policy” OR TI “Policies” OR AB “Policies” OR TI “Politics” OR AB “Politics” OR TI “Political” OR AB “Political” OR TI “Legislat*” OR AB “Legislat*” OR TI “Public health” OR AB “Public health” OR TI “Advocac*” OR AB “Advocac*” | 763,017 |
| S3 | TI “Competenc*” OR AB “Competenc*” OR TI “Experience*” OR AB “Experience*” OR TI “Capacit*” OR AB “Capacit*” OR TI “Capabilit*” OR AB “capabilit*” OR TI “Leadership” OR AB “Leadership” OR TI “abilit*” OR AB “abilit*” OR TI “Socialization” OR AB “Socialization” OR TI “Socialisation” OR AB “Socialisation” | 3,415,270 |
| S4 | TI “Empirical research” OR AB “Empirical research” OR TI “Focus group*”OR AB “Focus group*” OR TI “Semi-structured” OR AB “Semi-structured” OR TI “Semistructured” OR AB “Semistructured” OR TI “Unstructured” OR AB “Unstructured” OR TI “In-depth” OR AB “In-depth” OR TI “Indepth” OR AB “Indepth” OR TI “Face-to-face” OR AB “Face-to-face” OR TI “Grounded theory” OR AB “Grounded theory” OR TI “Phenomenolog*” OR AB “Phenomenolog*” OR TI “Ethnograph*” OR AB “Ethnograph*” OR TI “Field work” OR AB “Field work” | 346,386 |
| S5 | S1 AND S2 AND S3 AND S4 | **2,704** |
| **4. Embase (1966 – 31 December 2023)** | | |
| #1 | ‘Nurse*’:ti,ab OR ‘Registered nurse*’:ti,ab OR ‘Licensed nurse*’:ti,ab OR ‘Nursing professional’:ti,ab OR ‘Nursing profession’:ti,ab OR ‘midwifery’:ti,ab OR ‘midwife’:ti,ab OR ‘midwives’:ti,ab | 441,486 |
| #2 | ‘Policy’:ti,ab OR ‘Policies’:ti,ab OR ‘Politics’:ti,ab OR ‘Political’:ti,ab OR ‘Legislat*’:ti,ab OR ‘Public health’:ti,ab OR ‘Advocac*’:ti,ab | 902,779 |
| #3 | ‘Competenc*’:ti,ab OR ‘Experience*’:ti,ab OR ‘Capacit*’:ti,ab OR ‘Capabilit*’:ti,ab OR ‘Leadership’:ti,ab OR ‘abilit*’:ti,ab OR ‘Socialization’:ti,ab OR ‘Socialisation’:ti,ab | 4,506,503 |
| #4 | ‘Empirical research’:ti,ab OR ‘Focus group*’:ti,ab OR ‘Qualitative*’:ti,ab OR ‘Interview*’:ti,ab OR ‘Semi-structured’:ti,ab OR ‘Semistructured’:ti,ab OR ‘Unstructured’:ti,ab OR ‘In-depth’:ti,ab OR ‘Indepth’:ti,ab OR ‘Face-to-face’:ti,ab OR ‘Grounded theory’:ti,ab OR ‘Phenomenolog*’:ti,ab OR ‘Ethnograph*’:ti,ab OR ‘Fieldwork’:ti,ab OR ‘Field work’:ti,ab | 1,134,675 |
| #5 | #1 AND #2 AND #3 AND #4 | **5,195** |
| **5. Web of Science (1950 – 31 December 2023)** | | |
| #1 | TS=(‘Nurse*’ OR ‘Registered nurse*’ OR ‘Licensed nurse*’ OR ‘Nursing professional’ OR ‘Nursing profession’ OR ‘midwifery’ OR ‘midwife’ OR ‘midwives’) | 277,030 |
| #2 | TS=(‘Policy’ OR ‘Policies’ OR ‘Politics’ OR ‘Political’ OR ‘Legislat*’OR ‘Public health’ OR ‘Advocac*’) | 2,072,173 |
| #3 | TS=(‘Competenc*’ OR ‘Experience*’ OR ‘Capacit*’ OR ‘Capabilit*’ OR ‘Leadership’ OR ‘abilit*’ OR ‘‘Socialization’ OR ‘‘Socialisation’) | 6,794,250 |
| #4 | TS=(‘empirical research’ OR ’focus group*’ OR ’qualitative*’ OR ’interview*’ OR’semi-structured’ OR ’semistructured’ OR ’unstructured’ OR’in-depth’ OR ’indepth’ OR ’face-to-face’ OR’grounded theory’ OR ’phenomenolog*’ OR ’ethnograph*’ OR’fieldwork’ OR ’field work’) | 2,788,604 |
| #5 | #1 AND #2 AND #3 AND #4 | **6,355** |
| **6. SCOPUS (1981 – 31 December 2023)** | | |
| S1 | TI “Nurse*” OR AB “Nurse*” OR TI “Registered nurse*” OR AB “Registered nurse*” OR TI “Licensed nurse*” OR AB “Licensed nurse*” OR TI “Nursing professional” OR AB “Nursing professional” OR TI “Nursing profession” OR AB “Nursing profession” OR TI “Midwifery” OR AB “Midwifery” OR TI “Midwife” OR AB “Midwife” OR TI “Midwives” OR AB “Midwives” | 18 |
| S2 | TI “Policy” OR AB “Policy” OR TI “Policies” OR AB “Policies” OR TI “Politics” OR AB “Politics” OR TI “Political” OR AB “Political” OR TI “Legislat*” OR AB “Legislat*” OR TI “Public health” OR AB “Public health” OR TI “Advocac*” OR AB “Advocac*” | 74 |
| S3 | “Competenc*”[tiab] OR “Experience*”[tiab] OR “Capacit*”[tiab] OR “Capabilit*”[tiab] OR “Leadership”[tiab] OR “abilit*”[tiab] OR “Socialization”[tiab] OR “Socialisation”[tiab] | 38 |
| S4 | TI “Empirical research” OR AB “Empirical research” OR TI “Focus group*”OR AB “Focus group*” OR TI “Semi-structured” OR AB “Semi-structured” OR TI “Semistructured” OR AB “Semistructured” OR TI “Unstructured” OR AB “Unstructured” OR TI “In-depth” OR AB “In-depth” OR TI “Indepth” OR AB “Indepth” OR TI “Face-to-face” OR AB “Face-to-face” OR TI “Grounded theory” OR AB “Grounded theory” OR TI “Phenomenolog*” OR AB “Phenomenolog*” OR TI “Ethnograph*” OR AB “Ethnograph*” OR TI “Field work” OR AB “Field work” | 33 |
| S5 | 1 AND 2 AND 3 AND 4 | **18** |

**Supplementary table 3. Excluded Articles Based on Full-Text Review (*n* =116).**

Database and manual searches identified 17,810 studies, of which 27 individual studies were ultimately selected for meta-synthesis analysis. After the initial search, 8,353 duplicate articles were excluded, and 9,457 titles were reviewed. After excluding 68 non-English articles and 8,094 articles irrelevant to this study, the abstracts of 1,295 articles were reviewed. 1,152 articles that did not meet the selection criteria were excluded, and the full texts of the remaining 143 articles were reviewed. In the process, 116 articles on nursing practice competence, nursing policy evaluation, public health practice competence, political education, and policy analysis were excluded.

| **No** | **Authors, Year, Country** | **Journal** | **Title of article** | **Methodology** | **Research objective** | **Exclusion reason(s)** |
| --- | --- | --- | --- | --- | --- | --- |
| 1 | Ahmad  et al;  2023*;*  *Pakistan* | Rehman Journal of Health Sciences | Nurses’ involvement on policy making and implementation process regarding post coronary artery bypass grafting | Exploratory- qualitative study; Phenomenological research design | Exploring the experiences of nurses regarding involvement in policy making process about bypass patients | Nursing practice policy participation |
| 2 | Anifalaje;  2009;  United *Kingdom* | 3rd European Conference on Information Management and Evaluation IT Univ. | Governance Implications of Vertical Health Interventions on Health Information Systems Policy Implementation in Nigeria | Descriptive study | Understanding the dynamics between principals and their agents, how to influence policymakers, and analyzing the healthcare policy process | Healthcare policy analysis: Policy process |
| 33 | Antrobus & Kitson;  1999;  *United Kingdom* | Journal of advanced nursing | Nursing leadership: influencing and shaping health policy and nursing practice | Exploratory- qualitative study; Ethnographic approach | Examining critically contemporary nursing leadership within health policy | Nursing practice competence/ roles |
| 4 | Arabi  et al;  2014;  *Iran* | Iranian journal of nursing and midwifery research | Nurses’ policy influence: A concept analysis | Concept analysis: Walker & Avant approach | Clarifying the concept of nurses’ policy influence and to propose the definition of this concept | Concept analysis: Nurses’ policy influence |
| 5 | Asuquo: 2019;  *United Kingdom* | Journal of Nursing Management | Nurses’ leadership in research and policy in Nigeria: A myth or reality | Exploratory- qualitative study | Evaluating nurses' leadership in research and policy formulation | Analysing nurse's leadership in research and policy formulation |
| 6 | Bagnasco  et al;  2022;  *Italy* | Nurse Education in Practice | Core competencies for family and community nurses: A European e-Delphi study | e-Delphi study | Identifying the core competencies of family and community nurses | Nurses’ public health competence: Concept analysis |
| 7 | Barasteh  et al;  2021;  *Iran* | Frontiers in Public Health | Future Challenges of Nursing in Health System of Iran | Exploratory- qualitative study; Framework analysis method | Exploring the future challenges of nursing in the health | Challenges of nursing in the health |
| 8 | Baumgart  1980;  *Canada* | Canadian J of Nursing Research Archive | Nurses and political action: the legacy of sexism | Narrative qualitative study | Analysing nurses and the women's movement | Nurses’ political activity |
| 9 | Belita  et al;  2021;  *Canada* | PLoS One | Development and validation of a measure to assess evidence-informed decision-making competence in public health nursing | Development and content validation of a measurement tool | Developing and refining items for a new EIDM competence measure | Validating a tool to measure nurses' public health competencies |
| 10 | Benton;  2012;  *United States of America* | Online Journal of Issues in Nursing | Advocating Globally to Shape Policy and Strengthen Nursing's Influence | Descriptive study | Describing how nurses can advocate for the nursing profession by coordinating nursing activities | Policy perspective |
| 11 | Borges and Nascimento;  2004;  *Brazil* | Revista brasileira de enfermagem | The nurse in the decentralization process of the health system | Exploratory Qualitative Study | Describing of Urban Health Decentralization Processes and Nurse Deployment | Not an English paper |
| 12 | Benton  et al;  2017;  *Jordan* | International Nursing Review | An integrative review of pursing policy and political competence | Standard integrative review | Integrative review of nursing profession in policy and political processes | Integrative review |
| 13 | Bigbee  et al;  2010;  United States of America | Public Health Nursing | Public health nursing competency in a rural/frontier state | Cross-sectional descriptive design | Assessing the self-reported levels of competency among PHNs | Nurses’ public health competence/  Roles/  leadership |
| 14 | Blaauw  et al;  2014;  *South* Africa | Global health action | Nursing education reform in South Africa–lessons from a policy analysis study | Policy analysis framework | Undertaking a policy analysis study of the development of the new Nursing Qualifications Framework | Healthcare policy analysis |
| 15 | Blanchette;  2015;  The United States of America | University of Rhode Island | An exploratory study of the role of the town nurse | Descriptive, exploratory study | Exploring the roles usefulness in meeting the goals of the Patient Protection and Affordable Care Act | Nurses’ public health competence/  Roles/  leadership |
| 16 | Bou-Karroum  et al;  2017;  *Canada* | Implement-ation Science | Using media to impact health policy-making: an integrative systematic review | Qualitative methods, and case studies | Assessing the effects of media interventions on the health policy-making process | Systematic review |
| 17 | Brommeyer et al;  2023;  *Australia* | MIR Research Protocols | Developing Health Management Competency for Digital Health Transformation: Protocol for a Qualitative Study | Exploratory- qualitative study | Describing research that will contribute to developing the capability of health service managers | Nursing practice competence/ roles |
| 18 | Brooks;  2008;  *United Kingdom* | International Journal of Nursing Studies | Nursing and public participation in health: An ethnographic study of a patient council | Exploratory- qualitative study; Integrative ethnography | Exploring the relationship of the nursing profession to public participation | Nursing practice policy participation |
| 19 | Buchan & Calman;  2005;  *France* | OECD Health Working Papers | Skill-mix and policy change in the health workforce: nurses in advanced roles | Literature review | Describing research of policy change in the health workforce | Literature review |
| 20 | Cameron  et al;  2012;  *United Kingdom* | Journal of advanced nursing | Exploring leadership in community nursing teams | Exploratory descriptive design | Investigating how leadership is perceived in community nursing teams | Nurses’ public health competence/  Roles/  leadership |
| 21 | Canales & Drevdahl;  2022;  United States of America | Public Health Nursing | A Sisyphean task: Developing and revising public health nursing competencies | Literature review | psychometric evaluation research of the public health nursing competencies | Nurses’ public health competence/  Roles/  leadership |
| 22 | Chang  et al;  2008;  *Taiwan* | Journal of clinical nursing | Effects of an empowerment-based education program for public health nurses in Taiwan | Quasi-experimental design | Examining the effects of an empowerment-based education program | Public health/ policy nursing education |
| 23 | Chang  et al;  2003;  *Taiwan* | Journal of Nursing Research | Continuing education needs and barriers for public health nurses in Aboriginal townships in Taitung, Taiwan | Exploratory- qualitative study | Identifying the CE needs and barriers for 21 PHNs | Public health/ policy nursing education |
| 24 | Chiu  et al;  2021;  *Canada* | Policy, politics & nursing practice | Policy Advocacy and Nursing Organizations: A Scoping Review | Scoping Review | Examining the nature, scope, and domain of scholarly research focused on nursing organization and policy advocacy | Scoping Review |
| 25 | Choi;  2023;  *Republic of Korea* | Frontiers in Medicine | Enhancing nursing education to bolster nurse governance: insights from nurse managers | Exploratory- qualitative study; Thematic analysis | Exploring nursing education contents in undergraduate nursing programs | Enhancing nursing management education |
| 26 | Clancy  et al;  2013;  *Sweden* | Scandinavian Journal of Caring Sciences | Public health nursing and interprofessional collaboration in Norwegian municipalities: a questionnaire e study | Cross-sectional e-post questionnaire study | Examining collaboration relating to public health nursing | Not qualitative study |
| 27 | Clancy & Svensson;  2007;  *Sweden* | Nursing Philosophy | 'Faced' with responsibility: Levinasian ethics and challenges of responsibility in Norwegian public health nursing | Exploratory- qualitative study | Exploring the phenomenon based on the ethics of responsibility | Nurses’ public health competence/  Roles/  leadership |
| 28 | Coad  et al;  2018;  *United Kingdom* | Journal of Pediatric Oncology Nursing | Consult, Negotiate, and Involve: Evaluation of an Advanced Communication Skills Program for Health Care Professionals | Appreciative inquiry design | Sharing findings from a research evaluation of an advanced communicati-on skills training program | Nursing practice competence/ roles |
| 29 | Cross  et al;  2006;  *Canada* | Public Health Nursing | Development of the Public Health Nursing Competency Instrument | Development and validation of a measurement tool | Development and testing of an instrument to measure population-based public health nursing competencies | Nurses’ public health competence/  Roles/  leadership |
| 30 | Crowder;  2016;  United States of America | Journal of continuing education in nursing | Preparing Nurses to Communicate With the Media, Policy Makers, and the Public | Descriptive study | Describing as an active learning approach to developing nurses' critical communication skills | Public health/ policy nursing education |
| 31 | Currie  et al;  2010;  *United Kingdom* | Organization studies | Role Transition and the Interaction of Relational and Social Identity: New Nursing Roles in the English NHS | Exploratory- qualitative study | Examining the case of nurses in the English NHS | Nursing practice competence/ roles |
| 32 | Cusack  et al;  2018;  *Canada* | Journal of advanced nursing | Participatory action as a research method with public health nurses | Systematic research method | Describing participatory action research (PAR) as a preferred method | Public health research activities |
| 33 | Dahl & Crawford;  2018;  *United Kingdom* | ournal of interprofessional care | Perceptions of experiences with interprofessional collaboration in public health nursing: A qualitative analysis | Qualitative content analysis | Exploring public health nurses’ perceptions of their experiences | Experience of public health nursing |
| 34 | Dahl  et al:  2022;  *Norway* | Public Health Nursing | Norwegian public health nurses’ competence areas | Qualitative and comparative design | Exploring the competence areas of public health nurses | Nurses’ public health competence/  Roles/  leadership |
| 35 | Davis  et al:  1982;  United States of America | Journal of Nursing Administrat-ion | Leadership for expanding nursing influence on health policy | Checklists of specific activities | Enlarging their role in health policy making by increasing their political expertise | Literature review |
| 36 | Dekker  et al:  2020;  *Netherlands* | PCS–Politics, Culture and Socializatio-n | Political Socialization Theory, Research, and Application | Literature review | Focused on eight political socialisation agents | Literature review |
| 37 | Dollinger;  2007;  *United States of America* | Nursing Open | Health advocacy role performance of nurses in underserved populations: A grounded theory study | Exploratory- qualitative study; Grounded theory | Exploring how effectively nurses function as advocates in the federal health policy process | Healthcare policy analysis: Policy process |
| 38 | Ellenbecker & Edward;  2016;  United States of America | Policy, Politics, & Nursing Practice | Conducting nursing research to advance and inform health policy | Policy process framework | Providing evidence for informing and advancing health policies | Healthcare policy analysis: Policy process |
| 39 | Etowa  et al;  2023;  *Nigeria* | Int J Nurs Stud Adv | Experiences of nurses and midwives in policy development in low- and middle-income countries: Qualitative systematic review | Qualitative systematic review | A comprehensive analysis of qualitative evidence on the experiences of nurses and midwives participating in policy development | Qualitative systematic review |
| 40 | Fadlallah  et al;  2019;  *Thailand* | Health research policy and systems | Using narratives to impact health policy-making: a systematic review | Systematically review | Evidence on the use of narratives to impact the health policy-making process | Systematic review |
| 41 | Goolsby & Knestrick;  2017;  *United States of America* | Journal of the American Association of Nurse Practitioner | Effective professional networking | Exploratory- qualitative study | Combining nuggets from the literature with guidance based on the authors' combined experience in networking activities | Literature review |
| 42 | Goranitis  et al;  2014;  *United Kingdom* | Health Policy | Health policy making under information constraints: An evaluation of the policy responses to the economic crisis in Greece | Policy evaluation | Evaluating the health policy responses to the economic crisis | Healthcare policy evaluation |
| 43 | Gough & Walsh;  2000;  *United Kingdom* | Radcliffe Medical Press | Nurses and Nursing: Influencing Policy | Exploratory descriptive research | Describing nursing Influencing Policy | Healthcare policy evaluation |
| 44 | Hajbaghery & Salsali;  2005;  *Iran* | MC health services research | A model for empowerment of nursing in Iran | Grounded theory approach | Designing a model for empowering nurses | Nursing practice competence/ roles |
| 45 | Hajizadeh  et al:  2020;  *Iran* | BMC Nursing | A framework for nursing participation in health policy making: a systematic review | Systematic review | Designing a framework for nursing participation in health policy making | Systematic review |
| 46 | Hajizadeh  et al:  2021;  *Iran* | BMC nursing | Factors influencing nurses participation in the health policy-making process: a systematic review | Systematic review | Identifying factors influencing nurses participation in the health policy-making process | Systematic review |
| 47 | Hansen  et al;  2007;  *New Zealand* | Nursing Praxis in New Zealand | Public health nurses' views on their position within a changing health system | Mixed-method descriptive research study | Considering the context of public health nursing in New Zealand | Nurses’ public health competence/  Roles/  leadership |
| 48 | Harmon  et al;  2022;  United States of America | Public Health Nursing | Mixed-method analysis of the quad council competencies for public health nurses | Mixed-method descriptive research study | Evaluating the knowledge, skills, and attitudes (KSA) of public health nurses | Nurses’ public health competence/  Roles/  leadership |
| 49 | Harrington  et al;  2005;  United States of America | Policy, Politics, & Nursing Practice | Advanced nursing training in health policy: Designing and implementing a new program | Exploratory descriptive research | Providing an overview of a new master’s and doctoral educational program | Public health/ policy nursing education |
| 50 | Hauan  et al;  2023;  *Norway* | SAGE Open Nursing | Politically Engaged Mindset of Everyday Coping in Relation to Nursing Values: A Phenomenological-Hermeneutic Study of District Nurses' Experiences | Exploratory- qualitative study | Understanding how nurses practice care where everyday coping | Nursing practice competence/ roles |
| 51 | Haycock-Stuart  et al;  2010;  *Scotland* | The University of Edinburgh | Understanding leadership in community nursing in Scotland | Exploratory- qualitative study | Describing the leadership in community nursing | Nurses’ public health competence/  Roles/  leadership |
| 52 | Hewison;  2008;  *United Kingdom* | Policy, Politics, & Nursing Practice | Evidence-based policy: Implications for nursing and policy involvement | Exploratory- qualitative study | Involving taking a critical stance on the notion of evidence-based policy | Healthcare policy analysis |
| 53 | Hosseinzadegan et al;  2021;  *Iran* | Nursing ethics | Factors affecting nurses’ impact on social justice in the health system | Qualitative content analysis | Identifying factors affecting nurses’ participation in establishing social justice | Healthcare policy analysis |
| 54 | Hughes;  2010;  *United* *Kingdom* | Nursing praxis in New Zealand | The challenge of contributing to policy making in primary care: the gendered experiences and strategies of nurses | Exploratory- qualitative study | Development of the public health nursing role | Nurses’ public health competence/  Roles/  leadership |
| 55 | Jakeway  et al;  2006;  *United* States of *America* | Public Health Nursing | Developing population health competencies among public health nurses in Georgia | quantitative study:  Online  survey | Development of the public health nursing competence | Nurses’ public health competence/  Roles/  leadership |
| 56 | Joyce  et al;  2018;  United States of America | Public Health Nursing | Community/pub-lic health nursing faculty's knowledge, skills and attitudes of the Quad Council Competencies for Public Health Nurses | Mixed methods descriptive research | Evaluating the knowledge, skills, and attitudes of the 2011 QCC-PHN | Nurses’ public health competence/  Roles/  leadership |
| 57 | Juma et al.  2014;  Kenya | Nurs Res Pract. | Kenyan nurses involvement in national policy development | Mixed method: Qualitative and quantitative research | Understanding how nurses have participated in national policy processes in Kenya | Participants: Nurses and non-nurses included |
| 58 | Keepnews;  2006;  *United States of America* | Policy, politics & nursing practice | Bringing nursing leadership to shaping state policy: an interview with Virginia Trotter Betts | Interview with vice president of the American Nurses  Association | Developing nursing’s principles and positions on health care reform | Perspective interview in policy-making |
| 59 | Kelly’;  2011;  *Republic of Ireland* | Journal of Community Nursing | Preceptorship in public health nursing | Phenomenolo-gical research | Exploring the experience of being and Irish preceptor public health nurse | Public health/ policy nursing education |
| 60 | Kimiko  et al;  2017;  *Japan* | 5th Annual Worldwide Nursing Conference | Challenges and Solution Strategies for Public Health Nurses in Promoting Empowerment among Community Organization Activities in Japan | Exploratory- qualitative study | Strategies for Public Health Nurses in Promoting Empowerment | Nurses’ public health competence/  Roles/  leadership |
| 61 | Kisut  et al;  2022;  United States of America | BMC nursing | Competency assessment for community health nurses: a focus group expert panel discussion | Exploratory- qualitative study | Exploring perceptions and experiences of competencies assessment tool for CHN | Nurses’ public health competence/  Roles/  leadership |
| 62 | Kung and Rudner Lugo;  2015;  *United States of America* | Journal of the American Association of Nurse Practitioners | Political advocacy and practice barriers: a survey of Florida APRNs | Quantitative study: survey | Examining the relationships among demographics, years of RN and APRN experience | Not qualitative study: Quantitative study |
| 63 | Laari & Duma;  2023;  *Ghana* | Nursing Ethics | A qualitative inductive descriptive design by Creswell and Poth; qualitative content analysis | A qualitative inductive descriptive design;  content analysis | Exploring situations that thwart nurses from performing their health advocacy role | Barriers and facilitators for nurses' health advocacy |
| 64 | Larsen;  1980;  *Canada* | Canadian Journal of Nursing Research Archive | The Nursing Profession Viewed as a Political Pressure Group: Selected Review of the Literature | Literature review | Analyzing the extent to which organized nursing such as a political pressure group | Literature review |
| 65 | Lazarus & Lee;  2006;  United States of America | Policy, Politics, & Nursing Practice | Factoring consumers’ perspectives into policy decisions for nursing competence | Exploratory- qualitative study | Exploring the regulatory changes for nursing competence | Nurses’ public health competence/  Roles/  leadership |
| 66 | Lee & Choi;  2022;  *Republic* of *Korea* | Nurse Education Today | Educational strategies to encourage participation in health policy for nurses: A systematic review | Systematic literature review | Identifying evidence on the types of educational interventions | Systematic review |
| 67 | Lewinski & Simmons;  2018;  United States of America | The Journal of Continuing Education in Nursing | Nurse knowledge and engagement in health policy making: Findings from a pilot study | Quantitative research | Identifying nurses’ engagement in health policy making | Not qualitative study: Quantitative study |
| 68 | Maaitah & AbuAlRub;  2017;  *Jordan* | Revista latino-americana de enfermagem | Exploration of priority actions for strengthening role of nurses in achieving universal health coverage | Exploratory- qualitative study | Exploring priority actions for strengthening the role of Advanced Practice Nurses (APNs) | Nurses’ public health competence/  Roles/  leadership |
| 69 | Ma et al;  2021;  United States of America | Journal of Pain and Symptom Management | Playing Nice in the Sandbox: Lessons on Roles, Inter-disciplinary Collaboration, and Leadership from a VA National Improvement Intervention (F405D) | Exploratory- qualitative study | Identifying strategies that can increase health care system adoption of goals of care initiatives | Other profession' political competence |
| 70 | Maijala  et al;  2015;  *Finland* | Journal of clinical nursing | Identifying nurse practitioners' required case management competencies in health promotion practice in municipal public primary health care. A two-stage modified Delphi study | Two-stage modified Delphi study | Identifying NPs' required case management competencies | Nurses’ public health competence/  Roles/  leadership |
| 71 | McCready & Laperrière;  2023;  *Canada* | Journal of Advanced Nursing | The advocacy process in Canadian community health nursing: A collaborative ethnography | Collaborative ethnography | Exploring the process of advocacy in the context of community health nursing | Community-based  nurses' health advocacy (practice) |
| 72 | McGrath &. Walker;  1999;  *Australia* | Contempora-ry Nurse | Nurses' perception and experiences of advocacy | Exploratory- qualitative study: Thematic analysis | Discovering nurses’ perceptions and experiences of advocacy | Nurses’ patient advocacy |
| 73 | McKenna  et al;  2004;  *United Kingdom* | Journal of Nursing Management | Nurse leadership within primary care: the perceptions of community nurses, GPs, policy makers and members of the public | Exploratory- qualitative study:  Two round Delphi technique | seek the views of community nurses, general practitioners | Nurses’ public health competence/  Roles/  leadership |
| 74 | Miller;  2005;  United States of America | Social Science & Medicine | State health policy making determinants, theory, and methods: A synthesis | Systematic review and meta-synthesis | Synthesizing studies that use 50-state statistical techniques to model policy adoption | Systematic review |
| 75 | Myers;  2020;  United States of America | Nursing Clinics | Promoting population health: Nurse advocacy, policy making, and use of media | Descriptive study | Nurses’ advocacy, policy making, and use of media | Policy perspective/  Perspective  interview |
| 76 | Mills  et al;  2012;  *Canada* | Nursing Leadership (Toronto, Ont.) | Developing and sustaining leadership in public health nursing: findings from one British Columbia health authority | Mixed methods descriptive research | Developing clinical leadership among front-line public health nurses (PHNs | Nurses’ public health competence/  Roles/  leadership |
| 77 | Moriates  et al;  2015;  United States of America | Academic Medicine | Defining Competencies for Education in Health Care Value: Recommendations From the University of California, San Francisco Center for Healthcare Value Training Initiative | Exploratory- qualitative study | Defining foundational competencies of the health care value | Multidiscipli-nary profession's political competence |
| 78 | Najar & Hubbard;  2008;  *United States of America* | Policy, politics & nursing practice | The value of nurse leaders on federal advisory panels: experience with the Agency for Healthcare Research and Quality | Interview with six nationally known nurse leaders of National Advisory Committee | Discussing influence public policy | Policy perspective/  Perspective  interview |
| 79 | Nelson  et al;  2011;  *New Zealand* | Contempora-ry nurse | Adding value to stretched communities through nursing actions: The Wellington South Nursing Initiative | Descriptive study | Analysing nursing activities on Wellington South Nursing Initiative | Nurses’ public health competence/  Roles/  leadership |
| 80 | Okada  et al;  1997;  *Japan* | Japanese journal of public health | A study on competencies used by public health nurses in creating new health care systems in the community | Exploratory qualitative study | Clarifying the competencies used by public health nurses in creating new health care systems in the community | Unable to access |
| 81 | Parker;  2012;  United States of America | ills College | Nurse Leaders Working for Health Equity:  Experiences, Perspectives, and Insights | Exploratory qualitative study;  Grounded theory | Exploring the perspectives  of nurse leaders working for health equity | Nurses’ public health competence/  Roles/  leadership |
| 82 | Rabelo & Silva;  2022;  *Brazil* | Rev Bras Enferm | Let it not be that nursing that asks for silence: participation in social movements and sociopolitical-emancipatory knowledge | Exploratory qualitative study:  Foucault’s framework and descriptive analysis | Identifying expression of sociopolitical knowledge, based on nurses in social movements | Nurses’ social movements |
| 83 | Rains &. Barton-Kriese;  2001;  *United States of America* | Public Health Nursing | Developing political competence: a comparative study across disciplines | Exploratory- qualitative study | Comparing undergraduate nursing and political science students | Not nurses’ policy competence: Nursing students |
| 84 | Rasheed  et al;  2020;  *Pakistan* | Journal of Nursing Scholarship | Challenges, extent of involvement, and the impact of nurses’ involvement in politics and policy making in in last two decades: an integrative review | Integrative review | Determining  nurses' challenges, extent of involvement, and the impact of involvement in politics and policy making | Integrative review |
| 85 | Rezende  et al;  2020;  *Brazil* | Revista brasileira de enfermagem | Nurses' practice in quilombola communities: an interface between cultural and political competence | Exploratory- qualitative study | Understanding Family Health Strategy nurses' practices in the context of quilombola communities | Nurses’ public health competence/  Roles/  leadership |
| 86 | Richter  et al;  2013;  *Canada* | International Nursing Review | Nurses' engagement in AIDS policy development | Exploratory- qualitative study | Presenting findings related to the barriers and facilitators for nurses' engagement in policymaking | Barriers and facilitators for nurses' engagement in policymaking |
| 87 | Robertson & Baldwin; 2007;  *United States of America* | Clinical nurse specialist CNS | Advanced practice role characteristics of the community/public health nurse specialist | Exploratory- qualitative study | Describing the advanced practice role of nurses with master's degrees in community/public health nursing | Nurses’ public health competence/  Roles/  leadership |
| 88 | Saeki  et al;  2007;  *Japan* | Public Health Nursing | Factors associated with the professional competencies of public health nurses employed by local government agencies in Japan | Cross-sectional study | Clarifying some of the factors associated with the professional competencies of public health nurses (PHNs) | Not qualitative study |
| 89 | Safari  et al;  2020;  *Iran* | Journal of Nursing Research | The related factors of nurses’ participation and perceived benefits and barriers in health policy making | Cross-sectional descriptive study | Examining the factors related to the participation of nurses in the provision of health services | Policy perspective/  Perspective  interview |
| 90 | Salvage  et al;  2019;  *Switzerland* | International Nursing Review | Being effective at the top table: developing nurses' policy leadership competencies | Descriptive study | Developing their policy leadership competencies | Policy perspective/  Perspective  interview |
| 91 | Salvage & White;  2019  *Switzerland* | International Nursing Review | Nursing leadership and health policy: everybody's business | Descriptive study | Creating and strengthen strategic nursing leadership | Policy perspective/  Perspective  interview |
| 92 | Schofield  et al;  2011;  *Canada* | Journal of Nursing Research | Community Health Nursing Vision for 2020: Shaping the Future | Exploratory- qualitative study | Exploring development of a national vision for community health nursing | Nurses’ public health competence/  Roles/  leadership |
| 93 | Sharron & Crowder’  2016;  United States of America | The Journal of Continuing Education in Nursing | Preparing Nurses to Communicate With the Media, Policy Makers, and the Public | Exploratory- qualitative study | Using active learning strategies, educators facilitate nurses' ability to convey messages | Public health/ policy nursing education |
| 94 | Shillam & MacLean;  2018;  *United States of America* | Nursing Administration Quarterly | Leadership Influence: A Core Foundation for Advocacy | Systematic review | Presenting a framework for nurse administrators to use in developing direct care nurses in their leadership | Systematic review |
| 95 | Smolowitz  et al;  2015;  United States of America | Nursing Outlook | Role of the registered nurse in primary health care: meeting health care needs in the 21st century | Exploratory- qualitative study | The redesign of primary health care practice models to increase access to quality health care | Nurses’ public health competence/  Roles/  leadership |
| 96 | Spenceley  et al;  2006;  *Canada* | Policy, Politics, & Nursing Practice | The road less traveled: Nursing advocacy at the policy level | Exploratory- qualitative study | Reviewing the epistemological foundations of advocacy in nursing | Policy perspective/  Perspective  interview |
| 97 | Staebler  et al;  2017;  United States of America | Journal of Professional Nursing | Policy and political advocacy: Comparison study of nursing faculty to determine current practices, perceptions, and barriers to teaching health policy | Cross-sectional analysis | Understanding policy/advocacy concepts and methodology | Public health/ policy nursing education |
| 98 | Strudsholm &. Vollman;  2021;  *Canada* | Healthcare management forum | Public health leadership: Competencies to guide practice | Mixed methods descriptive research | Developing a set of interdisciplina-ry leadership competencies for seven public health disciplines | Nurses’ public health competence/  Roles/  leadership |
| 99 | Sudo  et al;  2023;  *Thailand* | Global Health & Medicine | The role of community nurse in the implementation of health policy for the elderly in Thailand | Exploratory- qualitative study: Thematic analysis | Identifying the role of community nurse in the implementation of Thai health policy for the elderly | Nurses’ public health competence/  Roles/  leadership |
| 100 | Sundean  et al;  2018;  United States of America | Nursing Outlook | The rationale for nurses on boards in the voices of nurses who serve | Explanatory sequential mixed methods design | Articulating the rationale for NOB in the voices of nurses who serve | Nursing practice competence/ roles |
| 101 | Swider  et al;  2013;  United States of America | Public Health Nursing | The quad council practice competencies for public health nursing | Descriptive study | Describing the most recent efforts by the Quad Council of Public Health Nursing organizations | Nurses’ public health competence/  Roles/  leadership |
| 102 | Thomas  et al;  2020;  United States of America | Policy, Politics, & Nursing Practice | How to engage nursing students in health policy: results of a survey assessing students’ competencies, experiences, interests, and values | Cross-sectional online survey study | Assessing nursing students’ perceptions of their competencies, experiences, levels of interest, and values concerning health policy | Not qualitative study: Quantitative study |
| 103 | Trought;  1993;  *United States of America* | University of Virginia | Policy making: State regulation of nursing practice. A study of the North Carolina Board of Nursing. University of Virginia | Exploratory- qualitative study:  Case study | Identifying overt factors that might  influence the policy making process | Healthcare Policy analysis |
| 104 | Turale, & Kunaviktikul;  2019;  *Thailand* | International nursing review | The contribution of nurses to health policy and advocacy requires leaders to provide training and mentorship | Descriptive study | Providing them with access to well-thought-out policy training programmes | Policy perspective/  Perspective  interview |
| 105 | Tveiten & Severinsson;  2004;  *Norway* | Nursing & Health Sciences | Client supervision: meaning and experiences from the perspective of Norwegian public health nurses | Exploratory- qualitative study | Examining the meaning and experiences of client supervision | Nurses’ public health competence/  Roles/  leadership |
| 106 | Vernon  et al;  2011;  *Australia, New Zealand* | International Nursing Review | Confidence in competence: legislation and nursing in New Zealand | Descriptive study | Identifying the competence under a new national regulatory regime | Nurses’ public health competence/  Roles/  leadership |
| 107 | Venturato  et al;  2007;  *Australia* | Journal of Nursing management | Nurses' experiences of practice and political reform in long-term aged care in Australia: Implications for the retention of nursing personnel | Exploratory- qualitative study:  Critical hermeneutic study | Exploring registered nurses’ experiences in long-term aged care in light of the political reform | Experience in public health/practice nursing |
| 108 | Wall;  2013;  United States of America | Nursing Outlook | The role of Catholic nurses in women's health care policy disputes: A historical study | Exploratory qualitative study | A Study on the Role of Catholic Nursing Sisters in Women's Health Policy | Historical study in healthcare policy |
| 109 | Waring  et al;  2023;  *United Kingdom* | Social Science & Medicine | Becoming active in the micro-politics of healthcare re-organisation: The identity work and political activation of doctors, nurses and managers | Exploratory- qualitative study: Narrative interview  study | Demonstration of political socialisation and activation between occupations | Multidiscipli-ne profession's political competence |
| 110 | Wolbers  et al;  2021;  *Netherlands* | Policy, Politics, & Nursing Practice | The Ambassador Project: Evaluating a Five-Year Nationwide Leadership Program to Bridge the gap Between Policy and District Nursing Practice | Mixed-methods  study | Evaluating from different perspectives the impact of this nationwide, five-year leadership program | Evaluation of nursing practice program |
| 111 | Worku  et al.  2020;  *Ethiopia* | Ethiopian Journal of Health Development | Development of core public health competencies for Ethiopia | Descriptive study | Describing the process and outcomes of the development of core public health competencies | Literature review |
| 112 | Yoshioka-Maeda;  et al;  2006;  *Japan* | International Journal of Nursing Studies | Tacit knowledge of public health nurses in identifying community health problems and need for new services: A case study | Exploratory- qualitative study:  Multiple-case study design | Exploring the tacit knowledge of public health and developing relevant new projects | Nurses’ public health competence/  Roles/  leadership |
| 113 | Young;  2009;  United States of America | Professional case management | Professional relationships and power dynamics between urban community-based nurses and social work case managers: advocacy in action | Community-based case management settings | Exploring how community-based case managers interface with their clients' healthcare providers and other community organizations | Nursing practice competence/ roles |
| 114 | Zachariadis  et al;  2013;  *United Kingdom* | BMJ open | Leadership of healthcare commissioning networks in England: a mixed-methods study on clinical commissioning groups | Mixed-method, multisite and case study research | Exploring the relational challenges for GP leaders setting up new network-centric commission organisations | Multidicinary profession's political competence/  leadership |
| 115 | Zerwekh;  1990;  United States of America | Seattle University | A qualitative description of the competencies of expert public health nurses | Phenomeno-logical perspective and constant comparative methodology | Describing the practice competencies of expert home visiting public health nurses | Nurses’ public health competence/  Roles/  leadership |
| 116 | Zeyana  et al;  2023;  United States of America | International Nursing Review | Implementing the nurse practitioner role in Oman: Implications for policymaking | Multiple situational analyses | Explaining Oman's national strategic plans, processes, challenges, opportunities | Nursing practice competence/ roles |

**Supplementary table 4. Selected articles list for analysis**

The 27 individual studies were included in the final analysis.

[1] C. Aarabi, A., Cheraghi, M. A., Ghiyasvandian, S., 2014. Factors effecting on involvement of nurse leaders in policy making for nursing in Iran: a qualitative study. International Journal of Advanced Nursing Studies, 3(1), 53-58.

[2] Barry, C. T., 1989. A descriptive study of the political socialization processes of nurses in specialized roles in the federal and state governments. George Mason University, Location.

[3] Cheraghi, M. A., Ghiyasvandian, S., Aarabi, A., 2015. Iranian nurses’ status in policymaking for nursing in health system: a qualitative content analysis. The open nursing journal, 9, 15.

[4] Chiu P, Thorne S, Schick‐Makaroff K, Cummings GG. Lessons from professional nursing associations’ policy advocacy responses to the COVID‐19 pandemic: an interpretive description. J Adv Nurs. 2023;79:2967-79.

[5] Deschaine, J. E., Schaffer, M. A., 2003. Strengthening the role of public health nurse leaders in policy development. Policy, Politics & Nursing Practice, vol. 4, no. 4, 266–274.

[6] DiGaudio, K. M., 1993. Nurses’ participation in policy making activities. State University of New York at Buffalo, Location..

[7] Ditlopo, P., Blaauw, D., Penn-Kekana, L., Rispel, L. C., 2014. Contestations and complexities of nurses’ participation in policy-making in South Africa. Global health action, 7(1), 25327.

[8] Donovan, D. J., Diers, D., Carryer, J., 2012. Perceptions of policy and political leadership in nursing in New Zealand. Nursing Praxis in New Zealand, vol. 28, no. 2, pp. 15–25.

[9] Gebbie, K. M., Wakefield, M., Kerfoot, K., 2000. Nursing and health policy. Journal of Nursing Scholarship, 32(3), 307-315.

[10] Hajizadeh, A., Zamanzadeh, V., Kakemam, E., Bahreini, R., & Khodayari-Zarnaq, R., 2021. Factors influencing nurses participation in the health policy-making process: a systematic review. BMC nursing, 20, 1-9.

[11] Hajizadeh, A., Zamanzadeh, V., Khodayari‐Zarnaq, R., 2021. Participation of nurse managers in the health policy process: A qualitative study of barriers and facilitators. International Nursing Review, vol. 68, no. 3, pp. 388–398.

[12] Hahn, J. A., 2019. The perceptions and experiences of national regulatory nurse leaders in advancing the advanced practice registered nurse compact policy agenda. Journal of the American Association of Nurse Practitioners, 31(4), 255-262.

[13] Han, N., 2020. Korean nurses’ participation in health care policy reform: A phenomenological study. Journal of Nursing Management, vol.28, pp. 1347–1355.

[14] Inayat, S., Younas, A., Andleeb, S., Rasheed, S. P., Ali, P., 2023. Enhancing nurses’ involvement in policy making:A qualitative study of nurse leaders. International Nursing Review, 70(3), 297-306.

[15] Kerschner, S. W., Cohen, J. A., 2002. Legislative decision making and health policy: A phenomenological study of state legislators and individual decision making. Policy, Politics, & Nursing Practice, 3(2), 118-128.

[16] Melo, C. M. M. D., Santos, T. A. D., 2007. Nurse's political participation in municipal Public Health Care System management. Texto & Contexto-Enfermagem, 16, 426-432.

[17] Michibayashi, C., Omote, S., Nakamura, M., Okamoto, R., Nakada, A. I., 2020. Competency model for public health nurses working on tobacco control in local governments in Japan: A qualitative study. Japan Journal of Nursing Science, 17(1), e12288.

[18] Moore, J. M., 2014. Circling the wagons and shooting inward: Understanding how nursing administrators approach the policy process (Doctoral dissertation, Indiana State University).

[19] Richter, M. S., Mill, J., Muller, C. E., Kahwa, E., Etowa, J., Dawkins, P., & Hepburn, C. 2013. Nurses' engagement in AIDS policy development. International Nursing Review, 60(1), 52-58.

[20] Shariff, N., 2015. A delphi survey of leadership attributes necessary for national nurse leaders’ participation in health policy development: An East African perspective. BMC Nursing, vol. 14, no. 1, pp. 13.

[21] Taylor, M. R., 2016. Impact of advocacy initiatives on nurses’ motivation to sustain momentum in public policy advocacy. Journal of Professional Nursing, vol. 32, no. 3, pp. 235–245.

[22] Waddell, A., Adams, J. M., Fawcett, J., 2017. Exploring nurse leaders' policy participation within the context of a nursing conceptual framework. Policy, Politics, & Nursing Practice, 18(4), 195-205.

[23] Warner, J. R., 2003. A phenomenological approach to political competence: Stories of nurse activists. Policy, Politics & Nursing Practice, vol. 4, no. 2, pp. 135–143.

[24] Wichaikhum, O., Abhicharttibutra, K., Nantsupawat, A., Kowitlawakul, Y., Kunaviktikul, W., 2020. Developing a strategic model of participation in policy development for nurses. International Nursing Review, vol. 67, no. 1, 11–18.

[25] Williams, T. T., 2018. Political advocacy in nursing: Perspectives from the field. The University of Alabama, Location.

[26] Wilson, A. A., 1997. Nursing effects in policy-making. The Union Institute.

[27] Wilson, D. M., Underwood, L., Kim, S., Olukotun, M., & Errasti-Ibarrondo, B. How and why nurses became involved in politics or political action, and the outcomes or impacts of this involvement. Nursing outlook, 70(1), 55-63, 2022.

**Supplementary table 5. The results of synthesis findings**

357 findings on nurses’ healthcare policy competence were derived from an analysis of quotations and individual study concepts. The findings were then synthesized into five factors, 15 themes, and 34 subthemes: Intrinsic Motivation (synthesized-themes: Awareness of healthcare issues, Sense of social responsibility, Commitment to policy advocacy), Healthcare Expertise synthesized-themes: Healthcare environments and systems, Healthcare knowledge, Healthcare information), Policy Development (synthesized-themes: Establishing strategic policy agenda, Conducting healthcare policy research, Developing policy and legislation), Political Skills (synthesized-themes: Communication, negotiation, and lobby, Networking and social coalition, Forming public opinion), Policy Intervention (synthesized-themes: Policy decision-making, Legislative engagement, Policy monitoring and improvement).

**Factors 1: Intrinsic Motivation**

| **Themes** | **Subthemes** | **Findings** | **References** |
| --- | --- | --- | --- |
| Awareness  of healthcare  issues | Perception of healthcare environment issues | Perception of social and healthcare problems | [2, 6, 8, 10, 13, 16, 20, 21, 23, 27] |
|  |  | Interest in social and health care issues | [2, 3, 6, 8, 10, 13, 16, 20, 21, 23-26] |
|  |  | Recognizing that law is the same for one and all | [27] |
|  |  | Wanting to protect future children from unhealthy environment | [17] |
|  |  | Historical perspective on nursing and politics | [21] |
|  |  | Awareness of the unjust healthcare events | [27] |
|  |  | Separation of personal feelings from political issues | [23] |
|  |  | Perception of excessive patient workload, nursing shortage, and lack of time | [19] |
|  | Recognition of healthcare policy problems | Grasping complex healthcare policy issues | [13, 21, 23, 25] |
|  |  | Recognize the limitations of policies that protect vulnerable population | [6, 17, 21, 25] |
|  |  | Recognizing healthcare policy problems about vulnerable populations | [6, 8, 21, 23, 25] |
|  |  | Lack of communication and sharing of policies | [19] |
|  |  | Recognizing the need to develop health policies that impact nursing practice to 'protect' nurses and patients | [19] |
|  |  | Perception of nurses' lack of participation in health policy development process | [19] |
| Sense  of social responsibility | Recognizing political responsibility as a nursing Professional | Responsibility and awareness of healthcare problems | [1, 6, 7, 13, 16, 17, 21, 23- 25] |
|  |  | Accepting accountability as the person in charge of healthcare policy reform | [17] |
|  |  | Taking responsibility to address biases against nurses | [13, 14, 22, 24, 25] |
|  |  | Nurses’ role and importance in policymaking | [13, 14, 24, 25, 27] |
|  |  | Politically engaged as a result of an experience that made them feel more responsible or recognize an opportunity | [9] |
|  | Interest in politics or healthcare policy | Perception of politics in nursing | [6, 7, 10 13, 21, 23- 25] |
|  |  | Interesting in politics from observing other nursing political activists | [6, 10, 11, 21, 25, 27] |
|  |  | Perception of politics beyond traditional stereotypes | [6, 10, 11, 21, 23, 25, 27] |
|  |  | Positive attitude towards policy intervention | [7] |
| Commitment  to policy advocacy | Sense of nursing values based on nursing identity | Pride in nursing profession | [6, 12, 21, 23, 25] |
|  |  | Professional identity | [6, 13, 16, 21, 23, 24, 25] |
|  |  | Recognizing that nursing is the essence of the public | [9] |
|  |  | Nurse's vision in anticipating future health problems | [6, 11, 13, 16, 20, 21, 23, 25, 27] |
|  |  | Sense of nursing values in influencing policy involvement | [6, 13, 16, 21, 23, 24, 25] |
|  |  | Unique advocacy perspective from nursing | [21] |
|  |  | Nurse's expectations of being heard | [23] |
|  |  | Respecting and embracing camaraderie and diversity | [9] |
|  |  | Firm belief that healthcare policy reform relies on the expertise of nurses | [17] |
|  | Willingness and persistence to drive policy change | Passion- driven advocacy | [6-8, 10, 13, 16, 21, 23, 25] |
|  |  | Sustaining advocacy through personal passion | [2, 6, 16, 20, 21, 22-24] |
|  |  | Perseverance through challenges | [2, 6, 16, 20, 21, 22-24] |
|  |  | Lifelong political activism | [21] |
|  |  | Initiative and proactivity in healthcare setting | [6, 10, 13, 21, 23-25] |
|  |  | Unwavering determination to remove barriers to healthcare policy reform | [13, 17, 23] |
|  |  | Strong motivation to pioneer and change policy reform | [17] |
|  |  | Maintaining momentum and discouragement avoidance | [23] |
|  |  | Consciously engaged politically because of passion for a particular cause | [9] |
|  |  | Professional self-empowerment | [14] |
|  |  | Persistence in achieving policy goals | [2, 6, 16, 20, 21, 22-24] |
|  |  | Sense of commitment | [2, 6, 16, 20, 21, 23, 24] |
|  |  | Persistence in advocacy | [2, 6, 16, 20, 21, 22-24] |

**Factors 2: Healthcare Expertise**

| **Themes** | **Subthemes** | **Findings** | **References** |
| --- | --- | --- | --- |
| Healthcare  environments and system | Identifying healthcare environments | Understanding the situation on healthcare environment | [2, 6, 8, 13, 17, 21, 23-25] |
|  |  | Ability to step back and assess situations | [16, 24] |
|  |  | Knowledge of health care environment and policies | [2, 6, 8, 13, 17, 21, 23-26] |
|  | Understanding healthcare systems and delivery models | Broad healthcare system understanding and perspective | [2, 6, 8, 13, 16, 17, 21, 23-26] |
|  |  | Understanding various healthcare sector expertise | [7, 8, 13, 23, 25] |
| Healthcare Knowledge | Understanding the healthcare policymaking process | Learning about policymaking processes and mechanisms | [2, 5, 6, 8, 9, 11, 13, 15, 16, 18-21, 23-26] |
|  |  | Unique knowledge and perspectives that should be impactful in government policy decisions | [16, 27] |
|  |  | Understanding the impact of health policy | [21, 23-25] |
|  |  | Value of public health expertise in policy development | [2, 5, 6, 8, 13, 20, 21, 24, 25] |
|  |  | Learning policy making through experience and fellowships | [6, 25] |
|  |  | Recognizing roles of the administration and sharing them with related organizations | [17] |
|  |  | Empowerment through policy knowledge | [14, 21, 23-25] |
|  |  | Understanding the direction of healthcare policy, social situations and changes at national and local level | [9, 17] |
|  |  | Nursing expertise as a credibility booster in policy process | [23, 25] |
|  |  | Cultivation of health policy knowledge | [2, 5, 6, 8, 9, 11, 13, 15, 16, 20, 21, 23-25] |
|  |  | Experiential policy learning and internship | [2, 9, 17] |
|  |  | Understanding that many stakeholders are involved in the policy process | [18] |
|  |  | Receiving mentorship in health policy to strengthen their skills | [2, 9, 17] |
|  | Understanding the complex nature of the political landscape and resource constraints | Understanding of political processes and structures | [2] |
|  |  | Recognizing the political elements that are part of the policy process | [2, 5, 6, 8, 13, 18-21, 24, 25] |
|  |  | Bringing nursing education and practice background skills to politics | [16, 27] |
|  |  | Understanding the complexity of the organizational structure, organizational processes, and multiple internal and external stakeholders | [2, 5, 6, 8, 13, 18-21, 24, 25] |
|  |  | Cultivating political knowledge among nurses | [24, 25] |
|  |  | Healthcare sector expertise related to politics | [20, 24] |
|  |  | Role of professionalism in healthcare policy | [13] |
|  |  | Empirical knowledge, Sociopolitical Knowledge and ethical knowledge | [22] |
|  |  | Learning from personal experience or by trial and error | [18] |
|  |  | Role of education, experience, and research in political knowledge acquisition | [6] |
| Healthcare Information | Gaining healthcare issues or problems | Identifying health policy problems or issues | [1, 3, 5, 13, 15, 17, 21, 24, 25] |
|  |  | Familiarizing policy issues | [9, 21, 23-25] |
|  |  | Addressing a variety of healthcare issues from a nurse practitioner perspective | [8-10, 13, 20, 21, 23, 25] |
|  |  | Creative and critical evaluation of social and health care issues | [26] |
|  | Collecting healthcare information that influences the policy process | Navigating key stakeholders and decision-makers that influence the policy process | [1, 3, 5, 13, 15, 17, 21, 24, 25] |
|  |  | Seeking political information | [1-3, 6, 8, 11, 13, 15, 16, 20, 21, 23-25] |
|  |  | Participating in workshops on healthcare policy reform to increase knowledge of the latest information, and trends in national policy | [17, 23-25] |
|  |  | Acquiring sufficient information about governmental or master policies | [1, 3, 5, 13, 15, 16, 21, 24, 25] |
|  |  | Responding to shifting needs, contexts, and evidence | [4] |
|  |  | Developing knowledge | [4] |

**Factors 3: Policy Development**

| **Themes** | **Subthemes** | **Findings** | **References** |
| --- | --- | --- | --- |
| Establishing strategic policy agendas | Setting the healthcare policy agenda and plan | Planning policy strategies and activities to influence health policy | [2, 6, 8, 9, 13, 20, 21, 24, 25] |
|  |  | Strategic approaches to policy advocacy, including policy needs, visionary thinking, and setting goals and priorities in policy contexts | [9, 17, 23-25] |
|  |  | Clarifying nursing perspective based on both opposition to and approval for healthcare policy reform | [17] |
|  |  | Being proactive and take initiative to formulate strategies of being involved at each stage of the policy development process | [2, 3, 6, 8, 9, 12, 13, 20, 21, 24, 25] |
|  |  | Evolution in policy development approach | [24] |
|  |  | Sharing experiences in health policy direction | [24] |
|  |  | Developing and establishing community-based measures for policy | [2, 3, 6, 8, 9, 13, 20, 21, 24, 25] |
|  |  | Incorporating support and information into existing healthcare projects | [17] |
|  |  | Narrowing down priority measures and positioning healthcare policy reform in the plan | [17] |
|  |  | Nurse's vision in anticipating future health problems | [1, 2, 3, 6, 8, 9, 12, 13, 20, 21, 24, 25] |
|  |  | Asking for policy advice from external experts as academic specialists | [17] |
|  |  | Discussing what might be the best policy when policy development | [18] |
|  |  | Increased awareness and importance of policy development | [5] |
|  |  | Evolution in policy development approach | [5] |
|  |  | Nature of the issue high priorities in health | [6] |
|  |  | Strategic health position | [16] |
|  |  | Continuously identify society's needs for nurses for policy participation | [1] |
|  |  | Initiatives specific to nurse researchers | [9] |
|  |  | Having professional credibility management skills | [9] |
|  | Considering the equity, efficiency, and feasibility of healthcare policy | Considering practical healthcare policy strategies in local situations, considering public nature, effectiveness, and feasibility | [17] |
|  |  | Setting clear but feasible goals for healthcare policy reform | [17] |
|  |  | Developing healthcare policy alternative in anticipation of the ripple effect | [17] |
|  |  | Thinking about cost-effectiveness and using existing health services where possible | [17] |
|  |  | Considering the municipality's policy on healthcare policy reform | [17] |
|  |  | Evaluating feasibility for effectiveness when choosing strategy | [1, 6, 25] |
|  |  | Evaluating short- and long-term effects | [15] |
| Conducting healthcare policy research | Analyzing and interpreting data sets based on critical thinking | Analyzing and interpreting policy documents, health statistics, and research findings | [5, 6, 8, 9, 11, 13, 16, 20, 21, 23-25] |
|  |  | Having critical thinking | [9, 17, 23-25] |
|  |  | Analyzing the data and the health effects of healthcare policy reform | [2, 6, 8, 13, 17, 20, 21, 24, 25] |
|  |  | Assessing social situations, changes, and new information on healthcare issues | [5, 6, 8, 11, 13, 16, 17, 20, 21, 23-25] |
|  |  | Connecting nursing policy research and practice | [9, 17] |
|  |  | Collecting and analysing large data sets to influence policy | [9] |
|  |  | Seeking frontline nurses’ opinions when developing unit organizational policies | [14] |
|  |  | Conducting policy research beyond nursing | [9, 13, 21, 24, 25] |
|  |  | Positioning policy reform in higher-level plans and collaborative research, and supporting it across whole organizations | [17] |
|  |  | Studying health policy problems and issues | [6, 10, 13, 16, 20, 21, 23-25] |
|  |  | Advancing policy research as a part of collaboration | [17] |
|  |  | Preparing salient research and data | [18] |
|  |  | Understanding data providing enough data so that others involved in the in the policy process | [18] |
|  |  | Providing data that is easy to understand with this statement | [18] |
|  |  | Collaborative nature of policy development | [5] |
|  |  | Critically evaluating information | [15, 25] |
|  |  | Data collection and policy agenda setting | [5, 6, 8, 11, 13, 16, 20, 21, 23-25] |
|  | Generating research evidence to influence health policies | Conducting healthcare research that generates evidence to inform and influence health policy | [5, 6, 8, 9, 11, 13, 21, 24, 25] |
|  |  | Tracking anticipated health legislation to forecast research needs | [9] |
|  |  | Research activities based on evidence | [17] |
|  |  | Having problems solving skills | [9] |
|  |  | Publishing survey results on healthcare policy reform alternative | [17] |
|  |  | Evidence-based policy creation | [5, 6, 8, 9, 11, 13, 18, 21, 24, 25] |
| Developing policy and legislation | Developing healthcare policy and legislation | Policy development or nursing-related law development | [2, 5, 6, 8, 11, 12, 13, 16, 20, 21, 23-26] |
|  |  | Direct involvement in health policy development | [5, 21, 24, 25] |
|  |  | Proposing operable policy recommendations | [2, 5, 6, 8, 13, 20, 21, 23-25] |
|  |  | Developing policy alternatives to improve public health and nurses’ right | [2, 6, 10, 11, 13, 20, 21, 23-25] |
|  |  | Evolution of policy development | [5] |
|  |  | Focusing on policy reform goals to make policy changes or develop policies | [9] |
|  |  | Developing and improving healthcare policy alternative | [2, 5, 6, 8, 11, 12, 13, 16, 17, 20, 21, 23-25] |
|  |  | Indicating policies that the public can agree | [17] |
|  |  | Promoting participation in health policy development | [10, 20, 25] |
|  | Disseminating evidence-based developed policies | Operating and utilizing policy alternatives based on research findings | [6, 13, 21, 23, 24, 25] |
|  |  | Creating and promotion easy-to-understand policy materials | [17] |
|  |  | Providing policymakers with the information they need to make good policy decisions | [9] |
|  |  | Sharing experiences in health policy direction | [16] |
|  |  | Delineating policy alternatives | [15] |
|  |  | Disseminating as the cascade effect in policy development | [6] |

**Factors 4: Political Skills**

| **Themes** | **Subthemes** | **Findings** | **References** |
| --- | --- | --- | --- |
| Communication, negotiation and lobbying | Clear articulation of the political perspective using effective listening, speaking, or writing | Clarity and effective communication | [3, 5, 6, 8, 11, 13, 17-21, 22-25] |
|  |  | keeping constant communication among stakeholders | [12, 13, 17, 22-25] |
|  |  | Having an open mind about and a positive attitude about who’s driving policy | [18] |
|  |  | Clear priorities and ability to communicate well with a diverse group of people representing different interests | [6, 9] |
|  |  | Inviting external experts to talk to local residents and stakeholders | [17] |
|  |  | Considering the opinions of healthcare policy reform promoters and opponents neutrally and fairly | [17] |
|  |  | An attitude that does not create enemies, emphasizes relationships and focuses on communication | [17] |
|  |  | Communication and interpersonal skills | [2, 5, 6, 8, 11, 13, 17-21, 22-25] |
|  |  | Being prepared to change views and not linger on mistakes | [17] |
|  |  | Understanding the enthusiasm of external experts who support healthcare policy reform | [17] |
|  |  | Message articulation and emotional involvement | [22] |
|  |  | Challenges in advocacy communication | [20, 21] |
|  |  | Communicate with people who support specific policies or initiatives | [18] |
|  |  | Modes of communication honest and openness communication throughout the policy process | [18] |
|  |  | Using of electronic communication | [18] |
|  |  | Considering how decisions are made by persons they may need to influence | [15] |
|  |  | Considering the effects of decision making on colleagues and other player | [15] |
|  |  | Ability to collaborate, build consensus, and communicate with people | [9] |
|  |  | Communicate their advocacy priorities and proposed policy solutions to policymakers, legislators, and other key stakeholders | [25] |
|  |  | Balance between assertiveness and communication skills | [16] |
|  |  | Being assertive in raising nursing concerns related to health care to policy makers | [20] |
|  |  | Articulating health issues of concern to nursing | [2, 5, 6, 8, 11, 13, 17-21, 22-25] |
|  |  | Communicate effectively be effective communicators who are able to articulate and disseminate health policy related issues – listening, speaking, writing | [6, 8, 11, 13, 20, 21, 23-25] |
|  | Persuasive communication with a variety of stakeholders and policymakers | Persuading policymakers or coordinate on controversial issues | [6, 8, 9, 13, 20, 21, 23-25] |
|  |  | Involving bosses, colleagues, and stakeholders from other departments | [17, 20, 21] |
|  |  | Persuasive communication with a variety of stakeholders and policymakers to promote the interests of the public and the nursing profession | [6, 8, 9, 13, 17, 20, 21, 23-25] |
|  |  | Gaining motivation from strong resistance by external stakeholders | [17] |
|  |  | Not getting caught up in stakeholders' lack of understanding of healthcare policy reform | [17] |
|  |  | Clinical experiences as a basis for persuasion | [23, 25] |
|  | Negotiation skills to resolve conflicts and compromise | Negotiation and compromise skills necessary to enact policy | [6, 8, 9, 11, 13, 15, 17, 20, 21, 23-26] |
|  |  | Developing negotiation skills to promote effective political interaction, resolve conflicts, and compromise when necessary | [6, 8, 9, 11, 13, 20, 21, 23-25] |
|  |  | Wanting to make progress while finding common ground with those resisting healthcare policy reform | [17] |
|  |  | Understanding stakeholder intentions and approaching them appropriately | [17] |
|  |  | Conveying the intent and policies of local government and negotiating a win-win relationship | [17] |
|  |  | Increasing understanding within the agency and aiming for consensus with policy makers | [17] |
|  |  | Making adjustments to maintain dialogue and good relationships and avoid conflict | [6, 8, 9, 11, 17] |
|  |  | Overcoming special interest conflicts in healthcare | [13, 21] |
|  |  | Negotiating with special interest groups | [12, 13] |
|  |  | Negotiation for healthcare policy reform | [18] |
|  |  | Skilled at influencing large groups of people | [9] |
|  |  | Leverage the value of health policy negotiation in a professional manner | [26] |
|  | Political lobbying through establishing effective channels with policymakers | Hiring lobbyists and meeting with the legislature on a regular basis attending committee meeting | [5, 6, 8, 12, 13, 21, 23, 25] |
|  |  | Efforts supported by lobbyists | [5, 6, 8, 13, 21, 23, 25] |
|  |  | Lobbying and advocating on behalf of nurses and the public to influence policy | [5, 6, 8, 9, 13, 21, 23, 25] |
|  |  | Promoting policy alternatives and lobby for legislation to become reality | [26] |
|  |  | Lobbying governments and decision makers | [4] |
| Networking and social coalition | Participating in nursing representative organizations and non-governmental organizations | Becoming active in nursing professional organizations | [6, 9, 13, 20, 21, 23-27] |
|  |  | Representative organization and serving or holding office | [9] |
|  |  | Incorporating health policy objectives into nursing professional organizations/clubs | [27] |
|  |  | Embedding policy roles and functions under various nursing societies | [14] |
|  |  | Perception of organizational roles | [6, 8, 13, 21, 23, 25] |
|  |  | Being assisted engagement process was required for each nurse to become involved in politics or political action | [24] |
|  |  | Source of knowledge and inspiration with the potential to involve and awake the interest of fellow nurses in political action | [24] |
|  |  | Support and cohesion while remaining alert and agile in the environment of forward momentum | [6, 12, 13, 17, 23, 25] |
|  |  | Eliminating differences in nurses' perceptions about the importance of healthcare policy reform | [17] |
|  |  | Internships in higher education and law doctoral programs and in the government affairs departments of major associations | [9] |
|  |  | Professional membership as a source of power | [6] |
|  |  | Impact of organized nursing on political socialization | [2] |
|  |  | Nursing profession's endorsement influence | [10] |
|  |  | Understanding the ideas, interests, and positions of broad network | [4] |
|  |  | Empowering members and stakeholders | [4] |
|  | Collaboration with professionals, various stakeholders, politicians, and civic groups | Expanding activities by strategically collaborating with stakeholders or interest groups | [6, 8, 13, 16, 17, 21-25] |
|  |  | Alignment and collaborative professional direction among nursing groups | [12] |
|  |  | Collaboration among nurse leaders, national health care organizations, and regulatory bodies | [6, 14] |
|  |  | Collaboration between frontline nurses and nurse leaders | [14, 17] |
|  |  | Deepening cooperative relationships with related divisions of other departments | [17] |
|  |  | Working indirectly with social and environmental approaches | [17] |
|  |  | Promoting common ground with stakeholders | [17] |
|  |  | Working with local volunteers and related organizations | [17, 18] |
|  |  | Value of interdisciplinary collaboration | [6, 8, 13, 16, 21, 23-25] |
|  |  | Developing the capacity to oversee financial budgeting and interdisciplinary collaboration, and maintaining competence | [18] |
|  |  | Relationship building in advocacy | [20, 21] |
|  |  | Desire to proceed rapidly, seeking understanding and cooperation as necessary | [17] |
|  |  | Working with community members, working with different organizations | [6, 8, 13, 16, 18, 21, 23-25] |
|  |  | Working in policy through contact with people | [9] |
|  |  | Involvement and interacting with individuals engaged in public policy | [2] |
|  |  | Interdisciplinary involvement | [16] |
|  |  | Having respect for others | [20] |
|  |  | Being effective in collaborating and cooperating within and outside the profession | [6, 8, 13, 16, 20, 21, 23-25] |
|  | Developing network platforms with professionals, civic groups, politicians, and legislators | Building networks and promoting effective communication to gain support for policies | [5, 6, 9-11, 13, 16-20, 21, 23- 25] |
|  |  | Developing and maintaining network platforms with professionals, politicians, and legislators that influence health policy | [5, 6, 8, 10, 11, 13, 16, 17, 20, 21, 23-25] |
|  |  | Engagement with political candidates for policy intervention | [13] |
|  |  | Networking and building social coalitions for policy influence | [5, 13] |
|  |  | Developing learning opportunities and information exchange sites to improve skills of stakeholders | [17] |
|  |  | Regimental organization | [13] |
|  |  | Unity from the various professional organizations | [12] |
|  |  | Support and confidence from professional groups | [21] |
|  |  | Influence of networking and relationships | [21] |
|  |  | Nursing professional support networks | [21] |
|  |  | Networking as vital for support and opportunities | [23] |
|  |  | Engagement in community and professional activities through network | [16] |
|  |  | Establishment of a decision-making system comprised of multidisciplinary professionals | [16] |
|  |  | Building and leveraging partnerships and  coalitions | [4] |
|  | Obtaining political or economic support, resources, and information through networking | Ability to mobilize support for policy actions from diverse perspectives | [9] |
|  |  | Using local social resources that are enthusiastic about healthcare policy reform | [17] |
|  |  | Securing economic support, resources, and informational materials for enhancing and implementing policies and initiatives | [17] |
|  |  | Allocation of money in policy and compared the securing of resources | [18] |
|  |  | Empower nurses through reflective and supportive mechanisms | [14] |
|  |  | Timing and Support for Health Policies | [6, 25] |
|  |  | Economic Support for Health Care Policies | [6] |
|  | Collective actions and a unified voice through social coalition | Collective actions to improve nursing image | [2, 5, 6, 14] |
|  |  | Impact of collective action through nursing representations | [2, 5, 6, 21, 23-25, 27] |
|  |  | Importance of a unified nursing voice | [1, 2, 5, 6, 8, 21, 23-25, 27] |
|  |  | Active participation in party political activities | [13, 26] |
|  |  | Gaining access to political candidates or political parties through endorsement activities | [26] |
|  |  | Various political activities to recognize the value of nursing | [26] |
|  |  | Opportunities for influence and participation in the policy process with external stakeholders | [18] |
|  |  | Collective effort and external support | [2, 5, 6, 21, 23-25] |
|  |  | Recognition of collective skills | [5] |
| Forming public opinion | Using various media to promote policies and rally public support | Making activities by publishing results through newspapers and academic societies | [5, 6, 9, 11, 13, 14, 17, 20, 21, 25] |
|  |  | Interviews for TV, radio, and the local newspaper and seeing your name in the paper, or being invited to talk to a powerful group | [25, 27] |
|  |  | Getting all that media attention for other political-action goals | [27] |
|  |  | Positive reception they received from the media and from individual or group political entities | [27] |
|  |  | The respect that getting from the media and from the public made it easier to keep going politically | [27] |
|  |  | Raising public awareness and opinion | [5, 6, 9, 11, 13, 14, 20, 21, 25, 26] |
|  |  | Utilizing media and networks for health policy development | [6, 13, 20, 21, 23-25] |
|  |  | Disseminating information widely combining multiple approaches | [17] |
|  |  | Publicity efforts | [13] |
|  |  | Including a variety of policy engagement resources in media that nurses encounter | [9] |
|  |  | Role of social media in skill development and networking | [21] |
|  |  | Improving the social image of nurses and putting trust in nurses | [14, 21, 24, 25] |
|  |  | Articulating and increasing public awareness of nursing duties and achievements | [21, 24, 25] |
|  |  | Choosing methods that make local residents and stakeholders more self-aware | [17] |
|  |  | Using existing fliers and teaching materials effectively | [17] |
|  |  | Using materials and educational posters incorporating messages about public | [17] |
|  |  | Explaining policies on healthcare policy reform alternative to local residents and stakeholders | [17] |
|  |  | Public awareness and advocacy for policy change | [13] |
|  |  | Public awareness and empathy | [13] |
|  |  | Increasing public awareness of nursing contributions | [24] |
|  |  | Participating in campaigns and run for office | [9, 13, 23, 25] |
|  |  | Providing guidance and direction to the general public and government decision makers | [4] |
|  |  | Maintaining public awareness | [4] |
|  | Operating conferences, policy workshops, or forums to share health issues and influence policy | Having the ability to clearly articulate health issues of concern to nursing at policy development forums/arena | [13, 20, 23] |
|  |  | Representation at health policy forums/seminars | [13, 24, 25] |
|  |  | Participation in policy development workshops | [19] |
|  |  | Participating and operating in forum where representatives of the political parties | [13, 14, 21, 23, 24, 27] |
|  |  | Taking the initiative to join policy forums | [13, 14, 21, 23, 27] |
|  |  | Encourage participation of skilled nurses at policy forums advocacy of nurses | [14] |
|  |  | Imparting of ideas, images, and information between internal members of an organization and associated external organizations | [18] |
|  |  | Sharing policy research findings with policymakers to generate interest | [9, 13, 21, 23, 27] |

**Factors 5: Policy Intervention**

| **Themes** | **Subthemes** | **Findings** | **References** |
| --- | --- | --- | --- |
| Policy decision-making | Policy engagement in governmental committees or health departments, and policy groups | Proactive actions to participate in local and national policymaking | [2, 6, 11, 13, 14, 16, 20, 21, 25, 27] |
|  |  | Being a powerful force in policy interventions based on nursing practice experience | [2, 6, 9, 11, 13, 14, 16, 20, 21, 25, 27] |
|  |  | Participation in health national assembly | [24, 25] |
|  |  | Communicating a proposed plan to policy makers | [2, 6, 10, 12, 13, 21, 23, 25] |
|  |  | Gathering team and beginning working through the policy process | [18] |
|  |  | Leading policy advocacy for nurses' rights and public health | [2, 6, 7, 9, 11, 13, 16, 20, 21, 25, 27] |
|  |  | Being appointed to a major government committee | [13, 24, 25, 27] |
|  |  | Creating an organizational system for policy reform | [17] |
|  |  | Need for nurse politicians for effective policy improvement | [13] |
|  |  | Influencing health policy | [2, 6, 10, 13, 16, 20, 21, 23-25] |
|  |  | Nurses' involvement in politics and policy making | [2, 6, 7, 10, 12, 13, 16, 20, 21, 23-25] |
|  |  | Receiving policy makers' approval and creating mechanisms | [17] |
|  |  | Involvement of in health policy courses and voice in policy-making | [2, 6, 7, 10, 13, 16, 20, 21, 23-25] |
|  |  | Force that drives change as the process of policy implementation | [18] |
|  |  | Normalization of challenges in policy work | [23] |
|  |  | Advocacy for social justice | [5] |
|  |  | Running health policy fellowships to get nurses involved in policy | [9] |
|  |  | Advocating for nursing perspectives in policymaking | [2, 6, 7, 9, 11, 13, 16, 20, 21, 25, 27] |
|  |  | Protecting and supporting nurses’ physical, mental, social, and economic welfare | [4] |
|  |  | Protecting the public | [4] |
|  |  | Sustaining and strengthening nursing  workforce capacity | [4] |
|  |  | Applying special skills and nursing process techniques to contribute to policy making at all levels, from the institutional to the national level | [9] |
|  |  | Influencing national policy decisions through participation in various political committees | [26] |
|  |  | Advocating with government to influence government policy | [26] |
|  |  | Role models and peer influence in policy engagement | [6] |
|  |  | Participation in legislative meetings, professional organization activities, and as members of community committees, boards, and task forces | [2, 3, 6, 10, 13, 21, 23, 25] |
|  |  | Leading change initiatives | [3, 16] |
|  |  | Seizing and leveraging opportunities for policy engagement | [1] |
|  |  | Working in a senior position or consulting in a health care government agency | [3] |
|  |  | Acquiring position through nurses' appointment in healthcare policy field | [3] |
|  |  | Actions in policy making process including letter writing and voting | [6, 13, 23-25] |
|  |  | Establishing and managing a specialist committee for healthcare policy reform | [2, 6, 10, 13, 17, 21, 23, 25] |
|  |  | Empowerment through policy engagement | [25] |
|  |  | Engagement with political election candidate | [2, 6, 10, 13, 21, 23, 25] |
|  | Creating more opportunities through successful policy decision-making experiences | Referencing successful experiences and good practice as a model | [2, 6, 8, 13, 17, 21, 23-25] |
|  |  | Preparing the basis of healthcare policy intervention and creating opportunities | [17] |
|  |  | Leading to opportunities to participate in other policy issues or policymaking | [2, 6, 8, 13, 21, 23-25] |
|  |  | Nurses' credibility increases with policy making, leading to inclusion in other policy making activities | [6] |
|  |  | Improving health systems and health  service delivery | [4] |
|  |  | Pioneering opportunities for intervention and delivering effective and locally-appropriate activities | [17] |
| Legislative engagement | Engagement with the government and the legislation process | Participation in legislative advocacy, including testifying before legislative bodies or engaging with policymakers | [2, 5, 6, 10, 12, 13, 16, 20, 21, 23-25] |
|  |  | Finding a supportive legislative leader for policy agenda forward | [12] |
|  |  | Ability to influence legislation | [2, 5, 6, 10, 13, 16, 20, 21, 23-26] |
|  |  | Engagement with government and legislation process | [2, 5, 6, 10, 13, 16, 20, 21, 23-25] |
|  |  | Visibility and integration of nursing into legislative work | [23, 25] |
|  |  | building political capacity and confidence as a legislator | [27] |
|  |  | Communicating nursing perspectives to health policy decision makers | [19] |
|  |  | Leading legislative advocacy for nurses' rights and benefits | [2, 5, 6, 10, 13, 16, 20, 21, 23-25] |
|  |  | Advancing from easy goals to more difficult ones by lowering hurdles instead of making new laws | [17] |
|  |  | Engagement with government and legislation process | [25] |
|  |  | Legislative Success | [13] |
|  |  | Legislator engagement | [3, 21] |
|  |  | Communicating a proposed plan to policy makers | [25] |
| Policy monitoring and improvement | Monitoring policy implementation process | Disseminating and establishing healthcare policy implementation throughout the community | [17] |
|  |  | Achieving and monitoring healthcare policy reform | [13, 17, 21] |
|  |  | Determining timing for policy interventions | [17, 25] |
|  |  | Ongoing work on healthcare policy implementation that does not end with a single event | [17] |
|  |  | Sharing accomplishments with local residents and stakeholders to raise motivation | [17] |
|  |  | Understanding and disseminating good cases in policy intervention | [17] |
|  |  | Developing locally appropriate activities based on good practice | [17] |
|  |  | Devising effective interventions with little resistance | [17] |
|  |  | Using response to initiatives as motivation for activities | [17] |
|  |  | Supervising whether health and medical policies are properly implemented | [19] |
|  |  | Monitoring health policies and taking corrective action when necessary | [19] |
|  | Evaluating and improving the quality of healthcare policy implementation | Correcting policy distortions during policy implementation during the implementation process | [13, 17, 23] |
|  |  | Evaluating healthcare policy implementation efforts at individual, group, and community levels | [17, 25] |
|  |  | Reflective and adaptive processes whereby nurses assess the outcomes of their advocacy efforts, learn from their experiences, and strategize for future advocacy initiatives | [21] |
|  |  | Improving initiatives based on evaluation results | [13, 17, 23] |
|  |  | Environment analysis and influence | [13, 23] |

**Supplementary table 6. Synthesis finding assessment_ GRADE CERQual evidence profile.**

The reliability of the qualitative evidence synthesis results was verified using the GRADE-CERQual assessment.

| **Summary of findings** | **Methodological limitation** | **Coherence** | **Adequacy** | **Relevance** | **CERQual assessment of confidence in the evidence** |
| --- | --- | --- | --- | --- | --- |
| Finding 1. Most nurse political activists emphasized the importance of motivation to engage in healthcare policy interventions and stated “intrinsic motivation” as one of the healthcare policy competencies required of nurses. “Intrinsic Motivation” competence includes “Awareness of healthcare issues” [13,18,20,36,37,40,42,45,47,49-51,53-56], which encompasses the perception of healthcare environment issues [13,18,20,36,37,40,42,45,47,49-51,53-56] and healthcare policy problems [20,40,42,45,49,51,53,55]. | Minor methodological limitations: 2/16 study with a lack of consideration of ethical issues | No concerns about the coherence of data | No concerns about adequacy of data (16 studies) | No concerns about the relevance of data (16 studies) | High confidence:  The two studies of moderate quality, with minor methodological limitations, high coherence, high relevance, and no concerns about data adequacy |
| Finding 2 It also embeds a “Sense of social responsibility” [12,13,18-20,35,40,41,43,45,47,51-55], which encompasses not only recognizing political responsibility as the nursing professional [13,19,20,35,40,41,43,45,47,51-55] but also interest in politics or healthcare policy [12,13,18,40,41,45,51,53-55]. | Minor methodological limitations: 1/17 study with a lack of consideration of ethical issues and 1/17 study with unclear evidence of reflexivity | No concerns about the coherence of data | No concerns about adequacy of data (17 studies) | No concerns about the relevance of data (17 studies) | High confidence:  The one study of moderate quality, with minor methodological limitations, high coherence, high relevance, and no concerns about data adequacy |
| Finding 3. Additionally, “Intrinsic Motivation” includes “Commitment to policy advocacy” [12,13,18,20,36,40-45,47,48,51,53-55], which implies a sense of nursing values based on nursing identity [12,13,20,40-45,47,48,51,53-55] and willingness and persistence to drive policy change [18,20,36,40-45,48,50-55] . | Minor methodological limitations: 2/17 study with a lack of consideration of ethical issues and 2/17 study with unclear evidence of reflexivity | No concerns about the coherence of data | No concerns about adequacy of data (17 studies) | No concerns about the relevance of data (17 studies) | High confidence:  The two studies of moderate quality, with minor methodological limitations, high coherence, high relevance, and no concerns about data adequacy |
| Finding 4. Most participants stated the need for “healthcare expertise” as a prerequisite competence for nurses to intervene in healthcare policy. “Healthcare Expertise” includes “Healthcare environments and systems” [20,36,40-42,45,47,51,53-56], which implies identifying healthcare environments [20,36,40,42,45,47,51,53-56] and understanding healthcare systems and delivery models [20,36,40-42,45,47,51,53-56]. | Minor methodological limitations: 1/13 study with a lack of consideration of ethical issues and 1/13 study with unclear evidence of reflexivity | No concerns about the coherence of data | No concerns about adequacy of data (13 studies) | No concerns about the relevance of data (13 studies) | High confidence:  The one study of moderate quality, with minor methodological limitations, high coherence, high relevance, and no concerns about data adequacy |
| Finding 5. It also implies a “Healthcare knowledge” [12,13,19,20,36,38-40,42,43,45, 47,48,50-56], which encompasses understanding the healthcare policymaking process [12,13,19,20,36,39,40,42,43,45,51,53-56] and understanding the complex nature of the political landscape and resourceful constraints [13,36,39,40,42,45,47,48,50-52,54,55]. . Furthermore, “Healthcare expertise” includes “Healthcare information” [12,18,20,35-40,42,43,45-47,50,51,53-56] , which implies gaining healthcare issues or problems [18,35,37,39,42,43,45-47,51,53-56] and collecting healthcare information that influences the policy process [12,20,35-37,38-40,42,45-47,50,51,53-55] . | Moderate methodological limitations: 3/24 study with a lack of consideration of ethical issues and 4/24 study with unclear evidence of reflexivity | No concerns about the coherence of data | No concerns about adequacy of data (24 studies) | No concerns about the relevance of data (24 studies) | Moderate confidence:  The four studies of moderate quality, with minor methodological limitations, high coherence, high relevance, and no concerns about data adequacy |
| Finding 6 Furthermore, “Healthcare expertise” includes “Healthcare information” [12,18,20,35-40,42,43,45-47,50,51,53-56], which implies gaining healthcare issues or problems [18,35,37,39,42,43,45-47,51,53-56] and collecting healthcare information that influences the policy process [12,20,35-37,38-40,42,45-47,50,51,53-55]. | Moderate methodological limitations: 3/19 study with a lack of consideration of ethical issues and 4/19 study with unclear evidence of reflexivity | No concerns about coherence of data | No concerns about adequacy of data (19 studies) | No concerns about the relevance of data (19 studies) | Moderate confidence:  The four studies of moderate quality, with minor methodological limitations, high coherence, high relevance, and no concerns about data adequacy |
| Finding 7. Nurse political activists reported that strengthening “Policy Development” ability was essential. “Policy Development” includes “Establishing strategic policy agenda” [20,35-37,39,40,42-48,50,51,53-55] , which encompasses setting the healthcare policy agenda and plan [20,35-37,39,40,42-45,47,48,50,51,53-55] and considering the equity, efficiency, and feasibility of healthcare policy [20,35,40,46,55]. | Minor methodological limitations: 3/18 study with a lack of consideration of ethical issues and 3/18 study with unclear evidence of reflexivity | No concerns about coherence of data | No concerns about adequacy of data (18 studies) | No concerns about the relevance of data (18 studies) | Moderate confidence:  The three studies of moderate quality, with minor methodological limitations, high coherence, high relevance, and no concerns about data adequacy |
| Finding 8. It also embeds a “Conducting healthcare policy research” [12,18-20,36,39,40,42,43,45-48,51,53-55], which embraces analyzing and interpreting data sets based on critical thinking [12,18-20,36,40,42,43,45-47,53-55] and generating research evidence to influence health policy [12,20,39,40,42,43,45,48,51,54,55]. | Moderate methodological limitations: 3/19 study with a lack of consideration of ethical issues and 3/19 study with unclear evidence of reflexivity | No concerns about coherence of data | No concerns about adequacy of data (19 studies) | No concerns about the relevance of data (19 studies) | Moderate confidence:  The three studies of moderate quality, with minor methodological limitations, high coherence, high relevance, and no concerns about data adequacy |
| Finding 9. In addition, “Policy Development” includes “Developing policy and legislation” [12,18,20,36,39,40,42-45,47,48,50,51,53-56], which means developing healthcare policy and legislation [12,18,20,36,39,40,42-45,48,50,51,53-56] and disseminating evidence-based developed policies [20,40,43,45-47,51,53-55]. | Moderate methodological limitations: 3/18 study with a lack of consideration of ethical issues and 3/18 study with unclear evidence of reflexivity | No concerns about coherence of data | No concerns about adequacy of data (18 studies) | No concerns about the relevance of data (18 studies) | Moderate confidence:  The three studies of moderate quality, with moderate methodological limitations, high coherence, high relevance, and great concerns about data adequacy |
| Finding 10. Most participants emphasized the importance of “Political Skills” to facilitate nurses’ intervention in healthcare policy. “Political Skills” includes “Communication, negotiation, and lobbying” [12,20,36,37,39,40,42-46,48,50-56], which encompasses clear articulation of political perspective using effective listening, speaking, or writing [12,20,36,37,39,42-48,50-55], persuasive communication with a variety of stakeholders and policymakers [20,40,42,43,45,50,51,53-55], negotiation skills to resolve conflicts and compromise [12,20,40,42-46,48,50,51,53-56], and political lobbying through establishing effective channels with policymakers [38-40,42-45,51,53,55,56]. | Moderate methodological limitations: 3/21 study with a lack of consideration of ethical issues and 3/21 study with unclear evidence of reflexivity | No concerns about coherence of data | No concerns about adequacy of data (21 studies) | No concerns about the relevance of data (21 studies) | Moderate confidence:  The three studies of moderate quality, with moderate methodological limitations, high coherence, high relevance, and no concerns about data adequacy |
| Finding 11. It also embeds a “Networking and social coalition” [12,13,18-20,35,36,39,40,42-45,47,48,50,51,53-56], which embraces participating in nursing representative organizations and non-governmental organizations [13,18-20,36,40,42-45,50,51,53-56], collaboration with professionals, various stakeholders, politicians, and civic groups [19,20,36,40,42-45,47,48,50,51,53-55], developing network platforms with professionals, civic groups, politicians, and legislators [12,18 20,38-40,42-45,47,48,50,51,53-55], obtaining political or economic support, resources, and information through networking [19,20,40,43,48,55], and collective actions and a unified voice through social coalition [13,19,35,36,39,40,42,45,48,51,53-56]. | Moderate methodological limitations: 3/22 study with a lack of consideration of ethical issues and 4/22 study with unclear evidence of reflexivity | No concerns about coherence of data | No concerns about adequacy of data (22 studies) | No concerns about the relevance of data (22 studies) | Moderate confidence:  The four studies of moderate quality, with moderate methodological limitations, high coherence, high relevance, and high concerns about data adequacy |
| Finding 12. Additionally, “Political Skills” includes “Forming public opinion” [12,13,19,20,39,43,45,48,50,51,53-56], which implies utilizing various media to promote policies and rallying public support [12,13,19,20,38,39,43,45,50,51,53-56] and operating conferences, policy workshops, or forums to share health issues and influence policy [13,19,43,45,48-51,53-55]. | Minor methodological limitations: 2/17 study with a lack of consideration of ethical issues and 3/17 study with unclear evidence of reflexivity | No concerns about coherence of data | No concerns about adequacy of data (17 studies) | No concerns about the relevance of data (17 studies) | High confidence:  The three studies of moderate quality, with moderate methodological limitations, high coherence, high relevance, and high concerns about data adequacy |
| Finding 13. Nurse political activists mentioned that nurses’ healthcare policy competence requires “Policy Intervention” ability. “Policy Intervention” competence includes “Policy decision-making”[12,13,18-20,36,37,39-47,50,51,53-56], which encompasses policy engagement in governmental committees or health departments and policy groups [12,13,18-20,36-41,43-47,50,51,53-56] and creating more opportunities through successful policy decision-making experiences [20,36,38,40,42,45,51,53-55]. | Moderate methodological limitations: 3/24 study with a lack of consideration of ethical issues and 4/24 study with unclear evidence of reflexivity | No concerns about coherence of data | No concerns about adequacy of data (24 studies) | No concerns about the relevance of data (24 studies) | Moderate confidence:  The four studies of moderate quality, with moderate methodological limitations, high coherence, high relevance, and high concerns about data adequacy |
| Finding 14. It also embeds a “Legislative engagement,” which embraces engagement with government and legislation process [13,18,20,36, 37,39,40,44,45,47,49-51,53-56]. | Minor methodological limitations: 2/17 study with a lack of consideration of ethical issues and 1/16 study with unclear evidence of reflexivity | No concerns about coherence of data | No concerns about adequacy of data (17 studies) | No concerns about the relevance of data (17 studies) | High confidence:  The two studies of moderate quality, with moderate methodological limitations, high coherence, high relevance, and high concerns about data adequacy |
| Finding 15. Furthermore, “Policy Intervention” includes “Policy monitoring and improvement” [20,45,51,53,55,57], which means monitoring the policy implementation process [20,45,49,51,55] and evaluating and improving the quality of healthcare policy implementation [20,45,51,53,57]. | No methodological limitations: 5/5 studies with a clear evidence of reflexivity | No concerns about coherence of data | No concerns about adequacy of data (5 studies) | No concerns about the relevance of data (5 studies) | High confidence:  No methodological limitations, high coherence, high relevance, and no concerns about data adequacy |

**Supplementary table 7. eMERGe reporting criteria for meta-ethnography**

This study also complied with the eMERGe reporting criteria for meta-ethnography.

| **No.** | **Criteria Headings** | **Reporting Criteria** |
| --- | --- | --- |
| Phase 1—Selecting meta-ethnography and getting started | | |
| *Introduction* | | |
| 1 | Rationale and context for the meta-ethnography | Describe the gap in research or knowledge to be filled by the meta-ethnography, and the wider context of the meta-ethnography.  Section. Methods – page 4 |
| 2 | Aim(s) of the meta-ethnography | Describe the meta-ethnography aim(s)  Section. Methods – page 4 |
| 3 | Focus of the meta-ethnography | Describe the meta-ethnography review question(s) (or objectives)  Section. Methods – page 4 |
| 4 | Rationale for using meta-ethnography | Explain why meta-ethnography was considered the most appropriate qualitative synthesis methodology  Section. Methods – page 4 |
| Phase 2—Deciding what is relevant | | |
| *Methods* | | |
| 5 | Search strategy | Describe the rationale for the literature search strategy  Section. Methods – page 5, 6 |
| 6 | Search processes | Describe how the literature searching was carried out and by whom  Section. Methods – page 5, 6 |
| 7 | Selecting primary studies | Describe the process of study screening and selection, and who was involved  Section. Methods – page 6-8 |
| *Findings* | | |
| 8 | Outcome of study selection | Describe the results of study searches and screening  Section. Methods – page 6-8 |
| Phase 3—Reading included studies | | |
| *Methods* | | |
| 9 | Reading and data extraction approach | Describe the reading and data extraction method and processes  Section. Methods – page 13-15 |
| *Findings* | | |
| 10 | Presenting characteristics of included studies | Describe characteristics of the included studies  Section. Methods – page 6-11, Table 1 |
| Phase 4—Determining how studies are related | | |
| *Methods* | | |
| 11 | Process for determining how studies are related | Describe the methods and processes for determining how the included studies are related: - Which aspects of studies were compared AND - How the studies were compared  Section. Methods – page 6-12, Table 2 |
| *Findings* | | |
| 12 | Outcome of relating studies | Describe how studies relate to each other  Section. Synthesis Finding: page 15-20, Table 3 |
| Phase 5—Translating studies into one another | | |
| *Methods* | | |
| 13 | Process of translating studies | Describe the methods of translation**:** - Describe steps taken to preserve the context and meaning of the relationships between concepts within and across studies- Describe how the reciprocal and refutational translations were conducted- Describe how potential alternative interpretations or explanations were considered in the translations  Section. Methods –page 13-15 |
| *Findings* | | |
| 14 | Outcome of translation | Describe the interpretive findings of the translation.  Section. Synthesis Finding: page 15-20, Table 3 |
| Phase 6—Synthesizing translations | | |
| *Methods* | | |
| 15 | Synthesis process | Describe the methods used to develop overarching concepts (“synthesised translations”)Describe how potential alternative interpretations or explanations were considered in the synthesis  Section. Methods –page 13-15 |
| *Findings* | | |
| 16 | Outcome of synthesis process | Describe the new theory, conceptual framework, model, configuration, or interpretation of data developed from the synthesis  Section. Synthesis Finding: page 15-20, Table 3, Figure 2 |
| Phase 7—Expressing the synthesis | | |
| *Discussion* | | |
| 17 | Summary of findings | Summarize the main interpretive findings of the translation and synthesis and compare them to existing literature  Section. Discussion: page 26-29 |
| 18 | Strengths, limitations, and reflexivity | Reflect on and describe the strengths and limitations of the synthesis: - Methodological aspects—for example, describe how the synthesis findings were influenced by the nature of the included studies and how the meta-ethnography was conducted.- Reflexivity—for example, the impact of the research team on the synthesis findings  Section. Discussion: page 29 |
| 19 | Recommendations and conclusions | Describe the implications of the synthesis  Section. Conclusion: page 29, 30 |
